# Selection for production-related traits in *Pelargonium zonale*: improved design and analysis make all the difference

**DOI:** 10.1038/hortres.2017.4

**Published:** 2017-02-22

**Authors:** Heike Molenaar, Martin Glawe, Robert Boehm, Hans-Peter Piepho

**Affiliations:** 1 University of Hohenheim, Institute of Crop Science, Biostatistics Unit, Stuttgart 70599, Germany; 2 Klemm+Sohn GmbH & Co. KG, Stuttgart 70378, Germany

## Abstract

Ornamental plant variety improvement is limited by current phenotyping approaches and neglected use of experimental designs. The present study was conducted to show the benefits of using an experimental design and corresponding analysis in ornamental breeding regarding simulated response to selection in *Pelargonium zonale* for production-related traits. This required establishment of phenotyping protocols for root formation and stem cutting counts, with which 974 genotypes were assessed in a two-phase experimental design. The present paper evaluates this protocol. The possibility of varietal improvement through indirect selection on secondary traits such as branch count and flower count was assessed by genetic correlations. Simulated response to selection varied greatly, depending on the genotypic variances of the breeding population and traits. A varietal improvement of over 20% is possible for stem cutting count, root formation, branch count and flower count. In contrast, indirect selection of stem cutting count by branch count or flower count was found to be ineffective. The established phenotypic protocols and two-phase experimental designs are valuable tools for breeding of *P. zonale*.

## Introduction

The improvement of plant cultivars is reflected by the response to selection in a breeding program. Response to selection, in its simplest form, is defined as the difference between the mean phenotypic value of progenies of selected parents and the mean phenotypic value of the whole parental generation before selection.^1^ The better the phenotyping, the better is the response to selection.

For more than a century, selection in field crops has been evolving as phenotyping approaches and experimental design have improved. Today’s phenotyping techniques have broadened the focus from hand measurements of single-plant traits or destructive analysis towards non-destructive, holistic and high-throughput phenotyping in the field.^2^ Such phenotyping platforms include three-dimensional time-of-flight cameras, laser distance sensors, hyperspectral imaging, infrared thermometers, ultrasonic sensors and multi-spectral crop canopy sensors that can measure, for example, canopy temperature and spectral reflectance and plant crop height of wheat plots,^3^ biomass accumulation^4^ or can be used to investigate photosynthesis, nutrient uptake, and plant growth and development.^5^

By comparison, ornamental breeding still relies more heavily on the ‘breeder’s eye’ for judging if one cultivar is better than another. Reasons are: (i) phenotyping is limited largely to relatively easily scored traits like petal and leaf color or growth type (see International Union for the Protection of New Varieties of Plants (UPOV), TG/28/9 Corr.) and (ii) the traits phenotyped are relevant to plant variety protection and thus prioritized by ornamental breeders, in contrast to traits which are not listed by UPOV. There are other no less economically important production-related traits, however, for which, to our knowledge, UPOV does not provide protocols. Presently, these traits are improved through cultivation practices or post-harvest treatments and not through breeding efforts. For example, root growth is generally improved by application of hormones.^6^

Currently there are also large differences between crop and ornamental breeding with respect to the use of experimental designs and statistical analysis for phenotypic selection. Efforts to optimize designs in crop breeding date back more than a century.^7^ Improvements were first made accounting for the appropriate sample size to achieve the desired level of precision in estimates of effects and power of experiments. In addition, the need for replicates over time or within or over locations became clear and proposals were also made to randomize the allocation of treatments to experimental units.^7^ In 1930s, these findings were laid down in Fisher’s well-known book on experimental design.^8^ On the basis of these principles more complex designs were soon developed,^7^ and more recently two-phase experimental designs^9^ were introduced. Such designs are needed when an experiment is conducted in more than one phase. For example, in the first phase plants of a crop may be raised in a field experiment. In the second phase, samples from the field plots are then taken to the lab for analysis.^10^ Two-phase designs have the property that the observational unit changes from one phase to the next.^10^ Further, phases may overlap.^10^ By using two-phase experimental designs it is possible to account for environmental effects on experimental units in previous experimental phases, which might influence a response when measuring the trait in a later experimental phase. Typically, such designs are used in cereal breeding. In this respect again, ornamental breeding is still lagging behind, although two-phase experimental designs are highly suitable for breeding ornamentals. For example, in *Pelargonium zonale,* a mother stock is established to harvest stem cuttings in the first phase, whereas in the second phase the genotypes are tested for root formation by rooting harvested stem cuttings. Despite the two-phase nature of this experimental setup, two-phase experimental designs have not been used so far in ornamental breeding.

Our objectives for improving phenotypic selection in *P. zonale* breeding were: (i) to establish scoring protocols for production-related traits, (ii) to introduce the use of two-phase experimental designs in ornamental breeding practice; and (iii) to quantify the increase in effectiveness of selection due to the introduction of measures described under (i) and (ii) by simulating the expected response to selection for production-related traits.

## Materials and methods

### Current breeding trials

**Figure 1:**
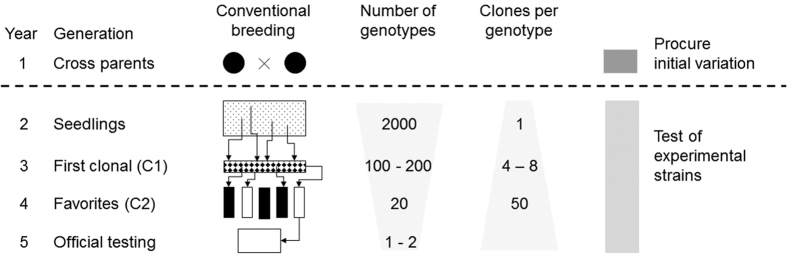
Current breeding scheme of *P. zonale*: from the intial parental crossing in year 1 to the official testing of the best lines in year 5, where the number of genotypes decreases, and in parallel, the number of clones per genotypes is increased.

Crosses of promising parental strains are made in year one of a breeding program. The 100–200 most promising candidates are selected from an unreplicated trial in year 2. Petal color, growth type and early prematurity are traits of primary interest. In year 3, selected candidates are tested under field conditions for assessment of petal color maintenance or drought tolerance, using four to eight clones of each candidate. In year 4 follows a production test (PT) accounting for real production conditions, which consists of two phases. In phase one (P1), the establishment of stock plants from which stem cuttings are harvested and the stem cutting count (SCC) is recorded. In phase two (P2), genotypes are assessed for rooting percentage, using the harvested stem cuttings of step one. Rooting percentage is defined as the number of rooted cuttings divided by the initially planted number of stem cuttings of one clone of a genotype in one tray. Up to 50 clones of one genotype are investigated. In the current protocol, a single clone of a genotype, placed on one tray, represents the observational unit of the trial, where clones of the same genotypes are placed next to each other in the greenhouses to have direct phenotypic comparisons. In statistical terms, real replicates of genotype are lacking as well as adherence to any other design principle, such as randomized allocation to experimental units, which would allow the application of statistically founded selection decisions. But efficient selection is of utmost importance in year 4, since selected clones are subjected to official variety testing (Figure 1).

### Experimental procedure of the current production test

To establish the stock plants, stem cuttings of selected genotypes are planted individually in paper pots (19 mm diameter, 33 mm height) filled with 80 % sterilized coco peat fibers and 20 % styroballs for aeration. The rooting takes 4 weeks under moderate climate conditions (15–28 °C) and irradiance between 20 and 25 klx depending on weather conditions. Fertigation starts in the third week after planting with a standard 2.5: 1 (N:K) menu containing the following nutrients (in mmol l^−1^): 21.0 NO_3_^−^, 3.5 $SO_{4}^{-2}$, 3.0 *H*_3_*PO*_4_, 1.4 $NH_{4}^{+}$, 9.0 K, 7.0 Ca, 3.3 Mg, 25.0 Fe, 6.0 Zn, 25.0 B, 2.0 Cu and 2.0 Mo. A sufficient amount of Mn is contained in the soil and made available to plants by keeping the pH level below 6.0. In week 4, rooted cuttings are then repotted in ~17.3 cm diameter bags with a volume of 3 l filled with 80 % (inert) pumice and 20 % coarse coco peat fibers to cultivate the stock plants. Stock plants are pinched once to stimulate branching and again afterwards if necessary. After 18 weeks of growth, stem cuttings are harvested and counted. Cuttings must be ⩽6 cm in length, have two to four leaves of which one is fully developed, and may not have flower buds or open flowers. To score genotypes for rooting percentage, all harvested stem cuttings of a genotype and different stock plants are planted in a column-wise fashion onto the same trays (Easypot, 25/39, 35 mm height, HAWITA Gruppe GmbH, Vechta, Germany, three rows with 13 paper pots each), where always a single stem cutting is planted per paper pot. The climate conditions are moderate: 18 °C temperature during planting and otherwise 18–24 °C and irradiance approximately 20 klx. Two hours after planting, plants are misted for 24 h, after which misting is reduced over a period of about 2 weeks depending on weather conditions. Spray misting is carried out every 16 s when irradiance levels exceeded 20 klx.

### A two-phase experimental design for *Pelargonium zonale* breeding

To improve the current PT, two experiments were conducted introducing two-phase experimental designs. Initially, the two phases of each of the two experiments were defined maintaining the context of the current PT steps: In P1, the cultivation of stock plants of genotypes, which was done in location 1, and in P2, the rooting of plant material, which was performed in location 2. Both phases took place in greenhouses and did not overlap. The cultivation procedures followed the current PT, whereas the planting manner was changed.

### Two-phase experiment I

Two-phase experiment (TPE) I was conducted in 2013/14. Five hundred genotypes were scored for SCC on eleven dates, flower count (FC) and branch count (BC) on two dates during P1 as well as for root formation (RF) on three dates during P2 (Table 1). Three hundred and fifty genotypes belonged to an internal collection and 150 were new breeds.

In the first phase, an α-design^11^ was used and generated by CycDesigN 4.0 (VSN-International, https://www.vsni.co.uk). The four cultivation tables in the greenhouse represented the four replicates. Each replicate in P1 comprised 167 incomplete blocks with three experimental units (EU1) each, except that one had only two EU1. On each EU1 a pair of stock plants was placed.

In the second phase, a conventional experimental design could not be used, because of fast quality decline of stem cuttings and therefore the necessity to work efficiently. However, to adhere to randomization, the packaging of stem cuttings for transfer from location 1 to location 2 was exploited.

Therefore, the total experimental space, represented by *m* rooting tables, was divided into four regions. The replicates were assigned systematically to the regions. Further, *t*=36 trays were laid out on each rooting table. On each tray there were 39 paper pots arranged in three rows with 13 paper pots each.

**Figure 2:**
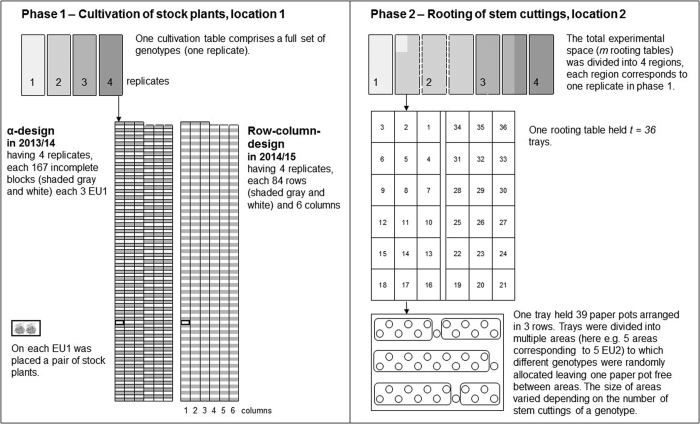
The two-phase experimental design intorduced in *P. zonale* breeding: P1, cultivation of stock plants for obtaining the SCC in location 1; P2, the rooting of stem cuttings to test the root formation in location 2. In P1, and α-design in 2013/14 and row-column design in 2014/15, were used. Each cultivation table represented on replicate having 500 planting positions arranged either in 167 incomplete blocks with three experimental units (EU1) each in 2013/14 or, in year 204/15 in 84 rows and six columns. On each EU1 a pair of stock plants of a genotype was placed in P1. In P2, the total experimental space represented by *m* rooting tables (at maximum 9) was divided into four regions to which the replicates were systematically assigned. Regions shaded in gray in rooting tables in P2 correspond to replicates shaded in gray of cultivation tables in P1. Eeach rooting table held 36 trays at maximum. One tray contained 39 paper pots arranged in three rows. The trays were divided into areas, representing an experimental unit in P2 (EU2), to which different genotypes were randomly allocated. The size of areas varied depending on the numbers of stem cuttings for a genotype. The planting of stem cuttings followed a row-wise order.

It is noted, that all trays of a replicate did not necessarily fit on one rooting table, indicated by regions shaded in gray in rooting tables in P2, which correspond to replicates shaded in the same gray of cultivation tables in P1 in Figure 2. Further, the incomplete blocks from P1 did not necessarily fit on a single tray in P2.

The trays were divided into areas, which represented the experimental units in P2 (EU2). In each area were planted all the cuttings for a genotype from the replicate. The size of an area varied depending on the number of stem cuttings for the genotype and replicate allocated to it.

Further, for each area, the pots were filled in row-wise order on a tray. One area follows on from the previous area subject to the restriction that all the paper pots for an area were on the same trays. One paper pot was left free between areas for a better differentiation of genotypes after 4 weeks rooting.

The genotypes were allocated randomly to the areas as mentioned above by exploiting the packaging order. Harvested stem cuttings of each genotype and replicate were packed in small bags, such that each bag contained all stem cuttings from EU1 in P1 and put into cartons. Genotypes within replicates of P1 were kept together. In location 2, small bags were randomly drawn out of the cartons and planted in areas. Thus, stem cuttings from each EU1 in P1 were allocated to exactly one EU2 in P2.

### Two-phase experiment II

TPE II was conducted in 2014/15 with 504 genotypes. One hundred and eighteen genotypes belonged to the internal collection and 356 to new breeds. In addition, 30 randomly chosen genotypes of TPE I were tested again. The SCC was assessed on five dates during P1 and RF was tested on four dates (Table 1). The experimental design in P1 of TPE II was modified to a resolvable row-column design to account better for a spatial trend detected in TPE I. The row-column design was generated using CycDesigN 4.0. The four replicates were represented by the four planting tables, where each replicate comprised six columns and 84 rows (Figure 2). In P2, the same approach was used as in TPE I in P2. The losses per genotype and the losses of stock plants were much higher than in TPE I.

### Phenotypic protocols

SCC was assessed as the number of stem cuttings per plant for each pair of stock plants (EU1) and genotype in P1. All stem cuttings were either observed by pinching or obtained at harvest time.

**Figure 3:**
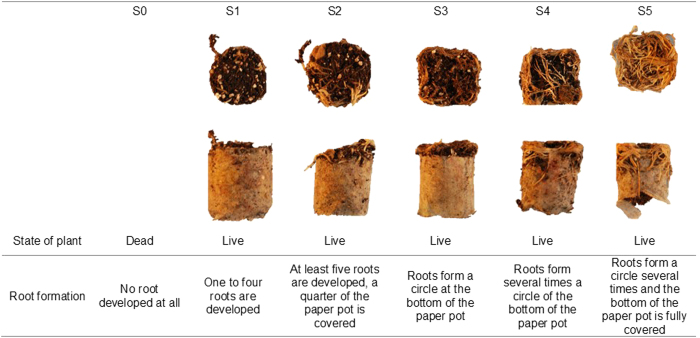
Ordinal categories of root formation ranging from S0 (dead) to S5 (extraordinary rooted).

The RF of stem cuttings of genotypes was described with six ordered categories after four weeks of growth (Figure 3) in P2. For each area, we counted the number of plants in categories S0 (dead) to S5 (extraordinary). From these counts we computed the sum of rooted cuttings assigned to S4 and S5, so that a single response value was obtained per area (EU2).

#### Secondary traits of SCC

FC was defined as the number of flowers per plant for each pair of stock plants (EU1) and genotype in P1 after eight and 12 weeks growth.

BC was defined as the number of all branches per plant for each pair of stock plants (EU1) and genotype evolved after 8 and 12 weeks growth.

### Statistical analysis

#### Single time-point analysis

SCC, FC, BC and the count of rooted cuttings assigned to categories (S4+S5) of RF were analyzed using a linear mixed model (LMM), where the randomization-based models in both phases were used for determining the terms in the model.^12^ The model notation followed by Piepho *et al.*,^13^ where the colon separates fixed effects on the left-hand side from the random effects on the right-hand side. The ‘dot’ operator (•) in a term A•B defines combinations of levels of its constituent factors A and B.

### Phase one model

To analyze SCC, BC and FC the model was successively setup as follows. The treatment model considering the randomized tier ^12^ was

(1)GEN,

where GEN denotes the genotypes (treatment factor). The randomization-based model considering the unrandomized tier^12^ was

(2)REP+REP.IB+REP.IB.PAIR,

where REP denotes the replicates represented by cultivation tables comprising a full set of genotypes, REP.IB the incomplete blocks nested within the replicates and REP.IB.PAIR, the EU1. Incomplete blocks were modeled as random since the block order was permuted during randomization. The full model obtained by combining the treatment and randomization-based model for design effects was

(3)GEN+REP:REP.IB+REP.IB.PAIR̲,

where the underlined term designates the residual error. The full model was augmented by a covariate, A, the number of stock plants per EU1 and genotype, because due to cultivation problems, some stock plants were missing at random. Further, a column (post-blocking) factor within replicates was added to better account for environmental effects. The model in analyzing SCC, BC and FC was

(4)A+GEN+REP:REP.IB+REP.COL+REP.IB.PAIR̲.

### Phase two model

To analyze the RF of stem cuttings assigned to categories (S4+S5) in P2, first the randomization-based model for P2 was set up as

(5)REGION+REGION.AREA,

where REGION denotes the experimental space to which systematically a replicate was assigned and REGION.AREA the EU2 to which the genotypes were randomly assigned. REP and REGION as well as REP.IB.PAIR and REGION.AREA were totally confounded terms as genotypes were kept together replicate-wise from P1 to P2 and the stem cuttings per experimental unit of P1 were held together and assigned to one area in P2. Thus, effects REGION and REGION.AREA do not need to be added explicitly to the model, as they are implicitly accounted for by the effects REP and REP.IB.PAIR, respectively. However, post-blocking was needed in P2, as variable environmental conditions between the rooting tables and between the trays occurred. To capture those variations, two post-blocking factors RTABLE and TRAY were defined. The former denotes rooting tables, each comprised of an incomplete set of genotypes, and the latter denotes trays, each comprised of multiple areas and which is nested within RTABLE. To exploit the inter-RTABLE and inter-TRAY information, both post-blocking factors were designated as random. The model for RF analysis was

(6)A+GEN+REP:REP.IB+REP.COL+RTABLE+RTABLE.TRAY+REP.IB.PAIR̲.

All statistical analysis was conducted with SAS 9.4 (SAS Institute Inc., Cary, NC, USA, 2014).

### Checking model assumptions

Independence of residuals, normal distribution of random effects (including the residual error) and variance homogeneity are important assumptions for LMM. To check these LMM assumptions, studentized residuals were investigated, which are independent of scale.^14^ A studentized residual is defined as $\frac{{\hat{e}}_{i}}{\sqrt{\mathrm{Var}[{\hat{e}}_{i}]}}$, where ${\hat{e}}_{i}$ is the *i*-th estimated raw residual and $\sqrt{\mathrm{Var}[{\hat{e}}_{i}]}$ the estimated s.d. of the *i*-th raw residual.^15^ To check normality, the studentized residuals were plotted against the normal scores in quantile–quantile plots (Q–Q-plots). To check for any unaccounted variance homogeneity, studentized residuals were plotted against the predicted value.^16^ Note that the LMM may entail a model allowing for heterogeneity of variance. If the model is well specified, the studentized residuals should display no remaining heterogeneity of variance. Normal distribution of random genotypic effects was checked using standardized best linear unbiased predictors (BLUPs)^17^ $\frac{{\hat{g}}_{j}}{\sqrt{\mathrm{Var}\left[{\hat{g}}_{j}\right]}}$, where ${\hat{g}}_{j}$ is the *j*-th estimated genotypic BLUP and $\mathrm{Var}\left[{\hat{g}}_{j}\right]$ its unconditional variance. These standardized BLUPs were plotted against the normal scores in Q–Q-plots.

### Model selection and fitting for repeated measurement analysis

For the traits SCC, BC, FC and counts of rooted cuttings assigned to (S4+S5) of RF repeated measurements were taken on the same plants at different harvest dates. A salient feature of repeated measurements is serial correlation among observations made on the same unit. To account for the repeated measurements nature of the data, the models (4) and (6) were expanded by a repeated factor T for time, by concatenating each factor with the repeated factor T as follows:^18,19^

(7)A+T+T.GEN+T.REP:T.REP.IB+T.REP.COL+T.REP.IB.PAIR̲

and

(8)A+T+T.GEN+T.REP:T.REP.IB+T.REP.COL+T.RTABLE+T.RTABLE.TRAY+T.REP.IB.PAIR̲.

For all random effects of model (7) serial correlations of observations were assumed. The best fitting variance–covariance structure was selected based on the smallest value of the Akaike information criterion (AIC).^20^ The AIC is defined as minus twice the REML log-likelihood plus twice the number of variance parameters.^21^ In model (8), serial correlations were only assumed for random effects defined for P1. The random effects defined for P2 were assumed to be independent, because at each single time-point genotypes were randomly allocated to areas. But still the repeated factor was concatenated with block factors of P2, because genotypes were systematically allocated to the same region, including the same rooting table, especially during RF assessment in TPE II, and seldom to the same area.

For selected variance–covariance structures, variance components of all model effects were estimated and used to predict the response to selection as well as to estimate the genotypic means for correlating estimates over experiments.

### Response to selection

Because data were unbalanced, the expected response to selection for SCC, FC, BC and RF was simulated using the fitted LMM ^22^ as

(9)Rq=∑iϵSqgi#(Sq)

and

(10)R=Q−1∑q=1QRq,

where Q is the number of simulation runs, R_q_ the predicted mean of the next generation, S_q_ the set of genotypes selected based on BLUPs of the true genetic values and #(S_q_) the size of the selected fraction. The central idea of this approach is to jointly simulate the genotypic effects (*g*_*i*_) and their BLUPs $\left({\hat{g}}_{i}\right)$ for a given experimental design. If we collect genetic effects and their BLUPs into a vector **w**, we may do a Cholesky decomposition of var(**w**) as var(**w**)=**Ω**=**ΓΓ**‘. To simulate **w** from a multivariate normal distribution with zero mean and variance–covariance matrix **Ω**, determined from the bits and pieces of the mixed model equations,^22^ a vector **z** of standard normal deviates is simulated that has the same length as **w**. A simulated realization of **w** is then obtained from **w**_**sim**_=**Γz**, so that the variance of the simulated data equals exactly the variance of the given data, var(**w**_**sim**_)=**ΓΓ**‘=**Ω**. The simulation was repeated 10 000 times. For each simulation run, the best values of BLUPs are selected to obtain the mean of the next generation based on the simulated true genetic values (*g*_*i*_). The predicted means of the next generation are then averaged over all 10 000 simulation runs to obtain the expected selection response.

### Genetic correlation between traits

Genotypic correlations^23^ between the totals of SCC, FC and BC were obtained in TPE I using the equation^24^

(11)rgij=σˆGijσˆGiσˆGj,

where ${\hat{\sigma }}_{Gij}$ is the estimated genotypic covariance between traits *i* and *j* and ${\hat{\sigma }}_{Gj}$ and ${\hat{\sigma }}_{Gj}$ are the estimated genotypic standard deviations for traits *i* and *j*, respectively. To estimate the genotypic variances and covariance, multivariate LMMs were fitted. In order to develop a multivariate model, model (4) was first extended by factor M, which identifies the three traits:

(12)M+M.REP.IB+M.REP.COL+M.REP+M.A:M.GEN+M.REP.IB.PAIR.̲

Nested structures between M and design factors were declared as fixed effects to alleviate the computational burden. The genotype factor was then considered as random. The vector *g*_*i*_ of genetic effects for the *i*-th genotype for the *T* different traits was assumed to be multivariate normal with *g_i_*~$MVN(0,\sum_{g})$, where ∑_*g*_ is given by ∑_*g*_=***D***_***g***_***R***_***g***_***D***_***g***_ with ***D***_***g***_, the diagonal matrix with genetic standard deviations for the *M* different traits on the diagonal and ***R***_***g***_ a *T*×*T* genotypic correlation matrix. Similarly, the vector *e*_*ij*_ of errors of the *j*-th observation on the *i*-th genotype was assumed to be multivariate normal with *e*_*ij*_~$MVN(0,\sum_{e})$, where ∑_*e*_=***D***_***e***_***R***_***e***_***D***_***e***_ with ***D***_***e***_ the diagonal matrix with standard deviations on the diagonal and ***R***_***e***_ a *T*×*T* error correlation matrix.

### Correlations of adjusted genotypic means over experiments

The precision assessment of the phenotyping approach based on the estimation of the Pearson correlation of the adjusted genotype means between the two experiments for genotypes assessed in both experiments for SCC and rooted cuttings assigned to categories (S4+S5) of RF.^25^ First, a repeated measurement analysis of each experiment was conducted selecting a variance–covariance structure for serial correlation of observations based on smallest AIC and then the genotype main effects for both traits were obtained. Second, the estimated genotype main effects were correlated between the TPE I and TPE II. The presence of genotype×time interaction will diminish the correlation, when genotype×time interaction is present.

## Results

### Checking model assumptions

The overall impression from plots of studentized residuals versus predicted values revealed that the variance–covariance model was appropriate but at the same time there was some departure from normality caused by outliers (Supplementary Figures 1 to 22). Removing outliers according to manually set trait-specific thresholds supported by the subject knowledge of the experiments (Table 2), approximate normality could be achieved and the plots of studentized residuals against the predicted means showed no non-normalities. Standardized genotypic BLUPs also showed approximate normality (Supplementary Figures 23 to 44).

### Model selection and fitting

The best model fit according to AIC was achieved for all traits with the unstructured variance–covariance structure for serial correlations of observations, except for RF of TPE I, where the smallest AIC was obtained for compound symmetry (Table 3). The variance components for selected variance–covariance structures presented in Table 4 were used to simulate the response to selection. Zero variance components of block factors mean that there was no correction due to those block factors during the estimation of effects. The largest variance for each trait is bold faced.

### Simulated response to selection

The simulated responses to selection for SCC, RF, FC and BC can be read from Table 5 as explained for SCC, at the first time-point of phenotyping, *l*=1, obtained in TPE I. The breeding population mean (*μ*) of SCC was 9.10 with a genotypic variance $\left(\sigma_{g}^{2}\right)$ of 3.98. When selecting the 40 best genotypes (*p*=40/*n*) out of the breeding population containing *n*=497 genotypes, the mean of the following generation would be increased by about three stem cuttings. Thus, the next-generation mean is expected to be 12.16 SCC. Numerical comparisons of predicted response to selection between time-points of the experiment and over experiments for the same traits are not meaningful, because *n* varied. The selected fraction *p*=*i/n* out of *n* has been defined by *i*=1, 5, 10, 20, 40 for all traits.

For SCC and RF, greater response to selection was observed during TPE I compared with TPE II as means and genotypic variance of these two breeding populations differed perceptibly. Selection of genotypes out of the breeding population of TPE I resulted in a population mean increase by two SCC at minimum in single time-point analysis when considering a selection intensity of *p*=40/*n*, whereas a selection of the best individual in the breeding population of TPE II would increase the population mean of the next generation by three SCC at maximum. When selecting for RF at a selection intensity of *p*=40/*n* in the breeding population of TPE I, the population mean can be doubled in the next generation in the best case, at time-point *l*=3. Selecting of genotypes in the breeding population of TPE II, the next-generation mean would be only increased by two-third of the breeding population mean. For BC and FC, which were phenotyped only during TPE I, similar results were found. At *p*=1/*n* and time-point *l*=2, the population mean of the following generation is increased by approximately six branches or flower counts per plant (Table 5).

### Genetic correlations of SCC, FC and BC

The obtained correlations between the totals SCC, FC and BC were in all cases in the low positive range. The total BC was found to have the highest genetic correlation with the total FC (*r*_*gij*_=0.2905). Marginally smaller was the genetic correlation between the total BC and the total SCC (*r*_*gij*_=0.2886), where the totals SCC and FC were found to have the smallest genetic correlation (*r*_*gij*_=0.1512).

### Pearson correlations of adjusted genotypic means over experiments

The Pearson correlation for SCC of adjusted genotypic means over the two experiments (*r*=0.37) was not found to be significantly different from zero (*P*=0.1301), whereas the Pearson correlation for rooted cuttings assigned to (S4+S5) of RF over the two experiments (*r*=0.56, *P*=0.0132) was approximately twice as high as for the SSC. The genotype×time interaction (GEN.T) was highly significant in both experiments for SCC (GEN.T: TPE I, *P*<0.0001 and TPE II, *P*=0.0088) and for RF (GEN.T: TPE I, *P*<0.0001 and TPE II, *P*<0.0001).

## Discussion

Our results show that there is great potential for varietal improvement of production-related traits in *P. zonale.* With the use of the developed phenotypic protocols, two-phase experimental design and its phase-specific analysis in the traits we analyzed, at least 20 % less stock plants would be needed to produce the same amount of stem cuttings as in the past. For example, given the test population mean and genotypic variance for SCC (TPE I, *l*=3), 10 stock plants were needed to produce in total 80 stem cuttings. After selection with the lowest selection pressure (*p*=40/*n*), only eight stock plants are needed to produce the same total (Table 5). This potential reduction of 20% less stock plants would mean in the final stage of stem cutting production that 250 000 stock plants can be saved resulting in a saving of 130 000 m^2^ greenhouse area, 50 000 m^3^ water, above 1 tonne of fertilizer as well as above 350 m^3^ substrate per year. By significantly improving genotypes for production-related traits the production becomes economically more efficient.

### The simulated response to selection

The prediction of response to selection assumes the same prerequisites as LMMs do.^22^ In checking those prerequisites, studentized residuals were investigated, suitable to detect outlying observations.^26^ Trait-specific thresholds were set based on the normal ranges observed in the greenhouse to remove outliers. In comparison to other methods for removing outliers, this is a simple method, and was preferred here, because little is improved by more complicated methods.^27^

The largest genotypic variances, in relation to the total variance, were obtained in analyses of SCC, FC and BC totals. As a result the largest simulated response to selection was obtained for these traits. The simulated response to selection in analyses of single time-points and repeated measurement were several fold lower for the same population. This was due to the relatively smaller genotypic variances obtained in analyses of single time-points and repeated measurements. Thereby, the simulated responses to selection of SCC obtained by repeated measurement analysis could be directly compared with the analyses of totals, where the simulated responses to selection obtained by repeated measurement analysis were multiplied by the number of observational time-points (*l*).

### Experimental designs in breeding practice

Experimental designs were developed which adapted the current ornamental breeding practice based on consideration of experimental design theory and practicality. For example, the approach in P2 of randomization was established to enable efficient working as well as maintain cutting quality and to provide flexibility for the sizes of areas within regions which varied according to the number of stem cuttings per genotype harvested. Biases of genotypic estimates could be avoided, which would have been caused without randomization due to heterogeneous conditions reflected by variance components of design effects.^28,29^

Further, post-blocking factors were introduced, which represented the physical units of production facilities especially in P2 allowing the consideration of sources of variation^30^ such as border effects caused by other cultivars, shades, heaters and fans in greenhouses.

The arrangement of clones was modified from current breeding practice for theoretical considerations. Clones are usually tested in a group-wise arrangement, the goal of which is to allow a simple scoring of the uniformity and stability of genotypes. However, we embedded the clones in the two-phase experimental layout as real replicates of genotypes (treatments) to allow estimation of variation^30^ and an unbiased estimation of genotypic effects, which is of more importance than simple scoring.

### Environmental effects and sources of errors

Variable environmental conditions are known to affect endogenous phytohormone levels in stock plants.^31^ This can influence the biosynthesis of leaf chlorophyll, color pigments and rooting of cuttings either positively or adversely.^31^ Blocking is a key strategy to control such variable conditions by making the conditions within blocks more equal than across blocks for testing treatments. In some cases, the residual error was not related at all to variable environmental conditions in the blocking factors, which were then estimated to be zero. These were in particular the replicate and row effects in analyzing SCC, BC and FC.

Some variable environmental conditions will not have been captured by the blocking structure and so will have been incorporated in the error. Some such environmental conditions were: first, varying seasonal temperatures in both experiments across single time-points influencing the regeneration capability. Seasonal temperature increase may increase leaf tissue dehydration levels of *P. zonale* during the rooting period,^32^ which is known to reduce the regeneration capability of stem cuttings.^33^ Second, varying day lengths across single time-points affecting the rooting. Day length is known to have an effect on rooting in other horticultural crops such as *Dahlia*.^34^ Furthermore, *P. zonale* is a short-day plant, which means its reproductive cycle, including vegetative and floral growth regulation, is affected by day length. Third, varying cutting storage length and conditions were present between harvest and planting. The standard storage duration of 4 days between harvest and planting has in our experience no negative effect on rooting. However, we noticed a negative effect on rooting and stock cultivation when the time between cooling chain and planting of stem cuttings lasted longer than 20 min and stem cuttings were subjected to temperatures over 25 °C when planting during summer periods. Serek *et al.*^35^ found an inhibition of rooting in terms of a reduced number and length of roots as well as reduced dry mass of roots of *P. zonale* cuttings after a short-term storage of already 3 days. In Serek’s^35^ study; however, a precise definition of the control treatment is lacking. Mutui *et al*.^36^ also found no adverse storage effect (4 days in the darkness) on rooting percentage, even though the length of roots and the number of roots per cutting were reduced. Fourth, varying pruning practices and watering are also likely to affect physiological processes. Pruning was variable due to alternating personnel who made different decisions regarding what constitutes a harvestable shoot. Watering varied in that there were differences in total water amount given between time-points, although within time-points, no spatial effects resulting from irrigation were observed. The effect of less water, or drought stress before phenotyping made roots poorly visible and differentiation difficult, which resulted in outlying observations especially in TPE I at *l*=2. An excess of water inhibited the development of roots resulting in a downgrading of RF of genotypes.

### Other considerations for selection

Selection on production-related traits should be reconsidered because the current indirect method of selection for SCC and FC, based on overall impression of the growth type and branching, is ineffective due to low correlation between these traits. One possibility is to count and assess stem cuttings for RF of selected genotypes in the seedling generation when they are vegetatively propagated for the first clonal generation (Figure 1). A selection of SCC and RF at single time-points has been found effective as there was sufficient genotypic variance (Table 5). Even better would be selection across single time-points, because the number of stem cuttings per plant increases with the plant’s age, and the ability to sustain stem cutting production over time is genotype-dependent. Therefore, the total SCC per genotype is a promising trait for selection.

Efficient selection of genotypes depends greatly on the phenotyping procedure. Phenotyping platforms for investigating biomass,^4^ which would be comparable to SCC, or X-ray computed tomography coupled with image-analyzing software packages^37^ to assess root formation were not affordable. Other, less costly, methods for phenotyping root traits, such as counting the number of roots or measuring their length.^35,36^ would have been too labor and time intensive for populations of the size considered here. Therefore, in P2, a scoring procedure for RF was established that extends the assessment of rooting percentage.^36^ In contrast to rooting percentage, defined as the proportion of rooted cuttings obtained from the total number of planted cuttings, RF allows the quality of each rooted cutting to be assessed. Further, rooting percentage was not found suitable for selection, since rooting percentage was generally high and varied little between genotypes. This agrees with results of Mutui *et al*.^36^ who found 100 % rooting in well-known *P. zonale* cultivars.

Throughputs of 125 stock plants in P1 and 5500 rooted cuttings in phase two per day were achieved. This makes the developed phenotyping protocol an effective and low-cost method comparable to high-throughput phenotyping procedures.

## Conclusion

With the help of the high-throughput phenotyping procedure developed and experimental design used in this study, genotypic variation could be effectively quantified, allowing varietal improvement of over 20 %.

Difficulties in implementing the experimental design were alleviated by a non-standard randomization approach observing experimental design principles.

We found that two-phase experimental designs in *P. zonale* breeding can reduce the error variances by accounting for phase-specific factors and increase the precision of estimates of phenotypic and genotypic effects, which positively affects the response to selection.

This study serves as a guideline to use experimental design, mixed models and response to selection in *P. zonale* breeding experiments. Further, it is expected that these techniques will be equally applicable to other species that involve similar phase-wise experimental setup.

## Figures and Tables

**Table 1:** Timeline of the TPE I and II in years 2013/14 and 2014/15, where in two phases genotypes were assessed for SCC, FC, BC and RF

*TPE*	*Year*	*Week*	*Phase*			
			*1*		*2*	
			*SCC*	*FC*	*BC*	*RF*
I	2013	41	x			
		43	x			
		46	x			
		50	x			
	2014	3	x			x
		7	x			
		9	x			x
		10	x			
		11	x			
		12	x			
		18	x			x
		26		x	x	
		34		x	x	
II	2014	35	x			x
		40	x			x
		45	x			x
		50	x			
	2015	3	x			x

Abbreviations: BC, branch count; FC, flower count; RF, root formation; SCC, stem cutting count; TPE, two-phase experiment.

**Table 2:** Thresholds for labeling outliers while residual outliers of trait analysis of SCC, RF (count of rooted cuttings assigned to *S4*+*S5*), BC and FC

*Trait*	*Threshold*
SCC	3.0
RF	3.25
BC	2.5
FC	3.0

Abbreviations: BC, branch count; FC, flower count; RF, root formation; SCC, stem cutting count.

**Table 3:** Model selection based on AIC for variance–covariance structures (VC, AR(1): first-order autoregressive model, CS, UN) for repeated measurement analysis of SCC, RF, FC and BC

*Trait*		*Variance–covariance structure*	*AIC*	
			*TPE I*	*TPE II*
SCC		VC	20319	11197
		AR(1)	20276	11147
		CS	20273	11100
		UN	**19815**^a^	**10817**
RF	Count of rooted cuttings assigned to categories (*S4*+*S5*)	VC	15902	11588
		AR(1)	15899	11541
		CS	**15897**	11518
		UN	15899	**11437**
FC		VC	3781.54	—
		AR(1)	3751.74	—
		CS	3751.7	—
		UN	**3741.61**	—
BC		VC	3398.55	—
		AR(1)	2822.18	—
		CS	2802.0	—
		UN	**2801.23**	—

Abbreviations: AIC, Akaike information criterion; BC, branch count; CS, compound symmetry; FC, flower count; RF, root formation; SCC, stem cutting count; TPE, two-phase experiments; UN, unstructured; VC, variance components.

a Smallest AIC is bold faced.

**Table 4:** Variance components of genotypic and design effects of single time-points (*l*) (GEN: genotypic variance, REP: replicate variance, REP.IB: row variance, REP.COL: column variance, RTABLE: rooting table variance, RTABLE.TRAY: tray variance, ERROR: residual error variance)

			*Phase 1*				*Phase 2*		
*TPE*	*Trait*	*l*	*GEN*	*REP*	*REP.IB*	*REP.COL*	*RTABLE*	*RTABLE.TRAY*	*ERROR*
I	SCC	1	3.98	0.05	0.77	0.46			**5.67**
		2	4.63	0.23	0.56	0.15			**9.01**
		3	1.93	0.1	0.12	0.02			**2.49**
		S_3_^a^	**26.62**	0	1.62	1.26			23.49
		S_11_^b^	**131.74**	0.53	0	8.87			117.38
		RP^c^	2.43	0.19	0	0.15			**6.9**
	RF	1	1.59	0.83	0	0.55	0.03	0.77	**6.7**
		2	2.17	0.97	0.06	0.03	1.41	0.49	**3.52**
		3	4.38	0.65	0.02	0.08	0.22	0.47	**6.26**
		RP^c^	1.74	0.99	0.03	0.17	0.26	0.62	**4.85**
	BC	1	**4.06**	0	0	0.49			3.34
		2	**4.49**	0	0	0.52			4.33
		S^d^	**27.67**	0	0	2.32			3.35
		RP^c^	5.61	0	0.72	0.31			**6.54**
	FC	1	3.67	0	0.03	0.95			**4.45**
		2	**7.14**	0	0.69	2.66			7.06
		S^d^	20.5	**53.79**	0	17.53			44.39
		RP^c^	2.86	0	0.001	0.63			**6.52**
II	SCC	1	0.08	0.04	0.1	0.03			**0.91**
		2	0.82	0	0.07	0.36			**2.19**
		3	0.3	1.1	0.27	0.09			**1.35**
		4	0.52	0.23	0.01	0.14			**3.81**
		S_4_^e^	4.03	3.19	0	0.7			**13.99**
		RP^c^	0.17	0.56	0	0.3			**2.32**
	RF	1	0.28	0.01	0.03	0.01	0.03	0.05	**1.26**
		2	0.79	0.78	0.06	0.24	0	0.18	**2.64**
		3	0.28	0.42	0.13	0.05	0.03	0.11	**1.93**
		4	0.83	0.41	0.08	0.25	0	0	**4.06**
		RP^c^	0.36	0.36	0.01	0.06	1.42	0.09	**2.63**

Abbreviations: AIC, Akaike information criterion; BC, branch count; CS, compound symmetry; FC, flower count; RF, counts of rooted cuttings assigned to S4+S5 of root formation; SCC, stem cutting count; TPE, two-phase experiment.

a Total over *l*=1, 2, 3 time-points.

b Total over *l*=1, …, 11 time-points.

c The variance components obtained by smallest AIC obtained by models (7) and (8) of repeated measurement analysis. In Supplementary Tables 1 to 6 are all estimated variance components obtained by by the repeated measurement analysis.

d Total over *l*=1, 2 time-points.

e Total over *l*=1, 2, 3, 4 time-points. The largest variance component for each trait is bold-faced.

**Table 5:** Predicted response to selection of the two TPE for assessed traits (SCC, RF: counts of rooted cuttings assigned to S4+S5 of root formation, FC, BC) for single time-point (*l*), total (S) and RP analysis for various selected fractions (*p*) for given population sizes (*n*)

*TPE*	*Trait*	*l*	*μ*	*p*					n
				*1/n*	*5/n*	*10/n*	*20/n*	*40/n*	
I	SCC	1	9.1	5.04	4.35	3.97	3.54	3.06	497
		2	6.46	5.21	4.5	4.11	3.67	3.17	496
		3	8.82	3.59	3.12	2.84	2.53	2.19	497
		S_3_^a^	24.46	13.93	12.05	10.99	9.82	8.48	497
		S_11_^b^^,^^c^	64.64	31.17	26.98	24.63	21.98	18.99	499
		RP^c^	8.12	2.36	1.92	1.68	1.41	1.13	497
	RF	1^d^	3.14	2.51	2.16	1.97	1.75	1.51	483
		2	4.09	3.58	3.09	2.82	2.51	2.17	485
		3	4.95	5.02	4.33	3.95	3.52	3.03	496
		RP^d^	3.69	2.52	2.18	1.99	1.77	1.53	497
	FC	1^c^	4.54	4.37	3.74	3.39	2.98	2.51	346
		2^c^	6.6	6.26	5.36	4.85	4.27	3.6	363
		S^c^^,^^e^	16.49	9.14	7.85	7.11	6.27	5.31	364
		RP	5.53	3.26	2.79	2.52	2.22	1.88	351
	BC	1	7.91	4.93	4.22	3.81	3.35	2.83	342
		2	8.04	6.09	5.24	4.76	4.23	3.61	347
		S^e^	15.74	14.72	12.58	11.37	9.99	8.41	336
		RP^f^	7.93	6.38	5.46	4.94	4.35	3.57	348
II	SCC	1	2.34	0.37	0.32	0.29	0.25	0.21	348
		2^c^	4.2	1.02	0.86	0.78	0.69	0.58	382
		3	3.84	1.25	1.08	0.98	0.86	0.73	372
		4	4.97	1.14	0.97	0.87	0.77	0.65	390
		S_4_^c^^,^^g^	15.6	3.93	3.34	3.01	2.64	2.23	390
		RP	3.85	0.78	0.66	0.6	0.53	0.45	394
	RF	1	1.66	0.95	0.81	0.73	0.64	0.54	349
		2^h^	3.02	1.74	1.48	1.34	1.18	1	373
		3	1.85	0.87	0.74	0.67	0.58	0.49	372
		4^h^^,^^i^	3.8	1.63	1.39	1.25	1.1	0.93	372
		RP	2.61	1.24	1.06	0.96	0.84	0.72	377

Abbreviations: AIC, Akaike information criterion; BC, branch count; CS, compound symmetry; FC, flower count; RF, root formation; RP, repeated measurement; SCC, stem cutting count; TPE, two-phase experiment; UN, unstructured; VC, variance components.

a Total over *l*=1, 2, 3 time-points.

b Total over *l*=1, …, 11 time-points.

c Estimates obtained without REP.IB in model (4).

d Estimates obtained without REP.IB in model (6).

e Total over *l*=1, 2 time-points.

f Estimates obtained without REP.COL in model (4).

g Total over *l*=1, 2, 3, 4 time-points.

h Estimates obtained without RTABLE in model (6).

i Estimates obtained without RTABLE.TRAY in model (6).

## References

[bib1] Falconer DS, Mackay TFC. Introduction to Quantitative genetics. Prentice Hall: London, UK, 1996.

[bib2] Walter A, Liebisch F, Hund A. Plant phenotyping: from bean weighing to image analysis. Plant Methods 2015; 11: 14. 25767559 10.1186/s13007-015-0056-8 PMC4357161

[bib3] Barker J, Zhang N, Sharon J, Steeves R, Wang X, Wei Y et al. Development of a field-based high-throughput mobile phenotyping platform. Comput Electron Agric 2016; 122: 74–85.

[bib4] Busemeyer L, Ruckelshausen A, Möller K, Melchinger AE, Alheit KV, Maurer HP et al. Precision phenotyping of biomass accumulation in triticale reveals temporal genetic patterns of regulation. Sci Rep 2016; 3: 1–6. 10.1038/srep02442 PMC3743059 23942574

[bib5] Panguluri SK, Kumar AA (ed.). Phenotyping for Plant Breeding. Springer: New York, NY. 2013.

[bib6] Mutui TM, Mibus H, Serek M. Effect of meta-topolin on leaf senescence and rooting in *Pelargonium*×hortorum cuttings. Postharvest Biol Technol 2012; 63: 107–110.

[bib7] Edmondson RN. Past developments and future opportunities in the design and analysis of crop experiments. J Agric Sci 2005; 143: 27–33.

[bib8] Fisher RA. The Design of Experiments. Edinburgh Oliver & Boyd: Edinburgh, Scottland, 1935.

[bib9] McIntyre GA. Design and analysis of two phase experiments. Biometrics 1995; 11: 822–828.

[bib10] Brien CJ, Harch BD, Correll RL, Bailey RA. Multiphase experiments with at least one later laboratory phase. I. Orthogonal designs. J Agric Biol Environ Stat 2011; 16: 422–450.

[bib11] Johnl JA, Williams ER. Cyclic and Computer Generated Designs. London Chapman & Hall: London, UK, 1995.

[bib12] Brien CJ, Demétrio CGB. Formulating mixed models for experiments, including longitudinal experiments. J Agric Biol Environ Stat 2009; 14: 253–280.

[bib13] Piepho HP, Büchse A, Emrich K. A hitchhiker’s guide to mixed models for randomized experiments. J Agron Crop Sci 2003; 189: 310–322.

[bib14] Cook DR, Weisberg S. Residuals and influence in regression. In: Cos DR, Hinkley DV(ed.). Monographs on Statistics and Applied Probability. Chapman & Hall: New York, NY. 1984.

[bib15] Littell RC, Milliken GA, Stroup WW, Wolfinger RD, Shabenberger O. SAS for mixed models. SAS Institute Inc: Cary, NC, 2006.

[bib16] Dufner J, Jensen U, Schuhmacher E. Statistic with SAS. Vieweg & Teubner Verlag: Tübingen. 2004, 171–180 German.

[bib17] Searle SR, Casella G, McCulloch CE. Variance Components. John Wiley & Sons: Hoboken, NJ, 1992.

[bib18] Piepho HP, Büchse A. A mixed modelling approach for randomized experiments with repeated measures. J Agron Crop Sci 2004; 247: 230–247.

[bib19] Piepho HP, Eckl T. Analysis of series of variety trials with perennial crops. Grass Forage Sci 2014; 69: 431–440.

[bib20] Akaike H. A new look at the statistical model identification. IEEE Trans Automat Contr 1974; 19: 716–723.

[bib21] Wolfinger R. Covariance structure selection in general mixed models. Commun Stat Simul Comput 1993; 22: 1079–1106.

[bib22] Piepho HP, Möhring J. Computing heritability and selection response from unbalanced plant breeding trials. Genetics 2007; 177: 1881–1888. 18039886 10.1534/genetics.107.074229 PMC2147938

[bib23] Piepho HP, Möhring J. On estimation of genotypic correlations and their standard errors by multivariate REML using the MIXED procedure of the SAS System. Crop Sci 2011; 51: 2449–2454.

[bib24] Holland JB. Estimating genotypic correlations and their standard errors using multivariate restricted maximum likelihood estimation with SAS Proc MIXED. Crop Sci 2006; 46: 642–654.

[bib25] Müller BU, Kleinknecht K, Möhring J, Piepho HP. Comparison of spatial models for sugar beet and barley trials. Crop Sci 2010; 50: 794–802.

[bib26] Schützenmeister A, Piepho HP. Residual analysis of linear mixed models using a simulation approach. Comput Stat Data Anal 2012; 56: 1405–1416.

[bib27] Bernal-Vasquez AM, Utz HF, Piepho HP. Outlier detection methods for generalized lattices: a case study on the transition from ANOVA to REML. Theor Appl Genet 2016; 129: 787–804. 26883044 10.1007/s00122-016-2666-6

[bib28] Piepho HP, Möhring J, Williams ER. Why randomize agricultural experiments? J Agron Crop Sci 2013; 199: 374–383.

[bib29] Stroup WW. Power analysis based on spatial effects mixed models: a tool for comparing design and analysis strategies in the presence of spatial variability. J Agric Biol Environ Stat 2002; 7: 491–511.

[bib30] Mead R, Curnow RN, Hasted AM. Statistical Methods in Agriculture and Experimental Biology. Chapman & Hall: Boca Raton, FL, 2002.

[bib31] Kelen M, Ozkan G. Relationships between rooting ability and changes of endogenous IAA and ABA during the rooting of hardwood cuttings of some grapevine rootstocks. Eur J Hortic Sci 2003; 68: 8–13.

[bib32] Kadner R, Druege U. Role of ethylene action in ethylene production and poststorage leaf senescence and survival of pelargonium cuttings. Plant Growth Regul 2004; 43: 187–196.

[bib33] Loach K. Water relations and adventitious rooting. In: Sankhla N (ed.). Adventitious Root Formation in Cuttings. Dioscorides Press: Portland, OR. 1989.

[bib34] Zimmermann PW, Hitchcock AE. Root formation and flowering of *Dahlia* cuttings when subjected to different day lengths. Bot Gaz 1929; 87: 1–13.

[bib35] Serek M, Prabucki A, Sisler EC, Andersen AS. Inhibitors of ethylene action affect final quality and rooting of cuttings before and after storahe. Hort Sci 1998; 33: 153–155.

[bib36] Mutui TM, Mibus H, Serek M. Effects of thidiazuron, ethylene, abscisic acid and dark storage on leaf yellowing and rooting of Pelargonium cuttings. J Hort Sci Biotechnol 2005; 80: 543–550.

[bib37] Kumar J, Kumar S, Pratap A (ed.). Phenomics in Crop Plants: Trends, Options and Limitations. Springer: New Delhi. 2015.

