# Supplementary material for: Selection for production-related traits in Pelargonium zonale: improved design and analysis make all the difference
Source: Hortic Res. 2017 Feb 22;4:17004–. doi: 10.1038/hortres.2017.4 (PMC5321157; doi:10.1038/hortres.2017.4)

## Slide 1
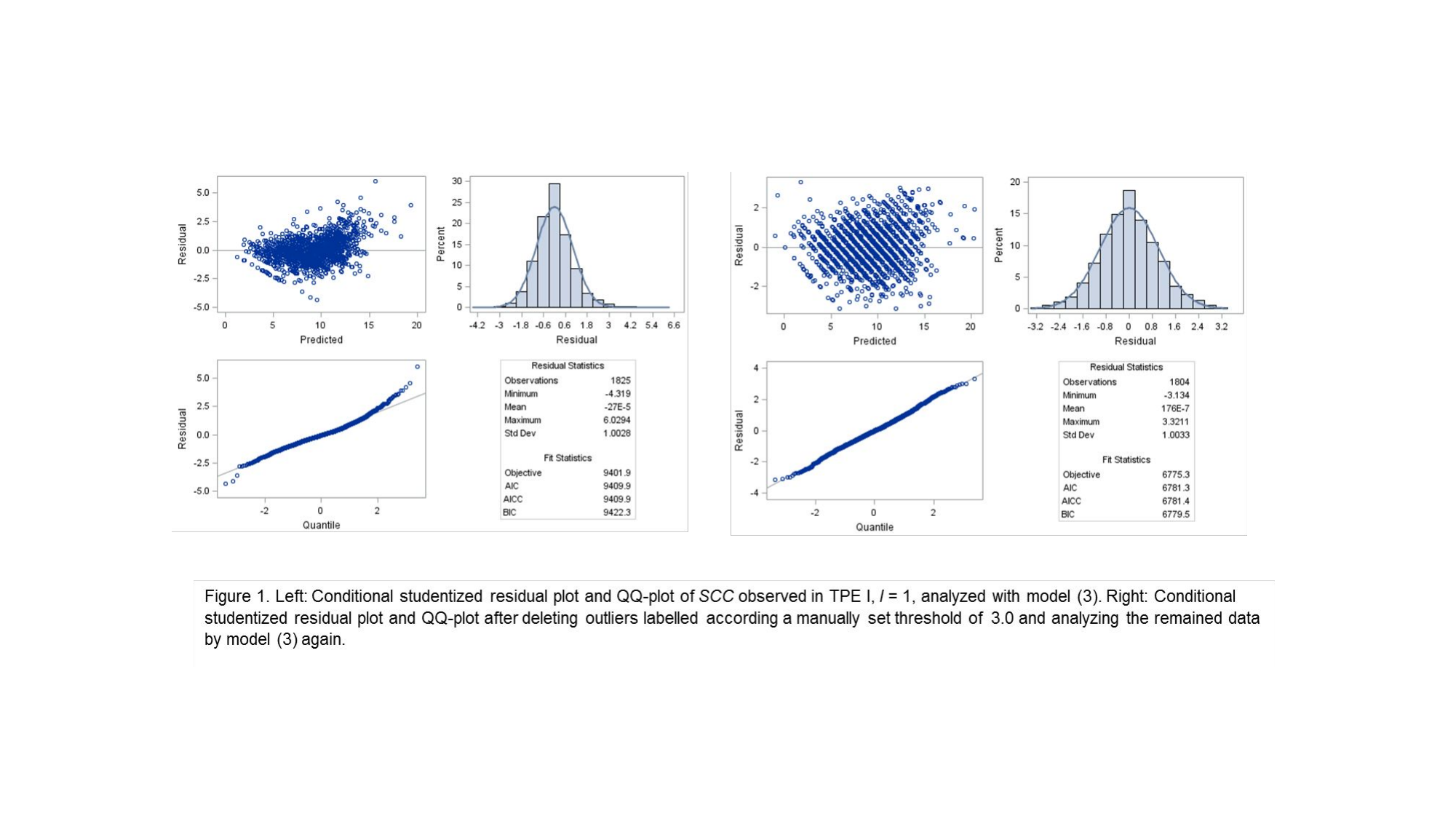

## Slide 2
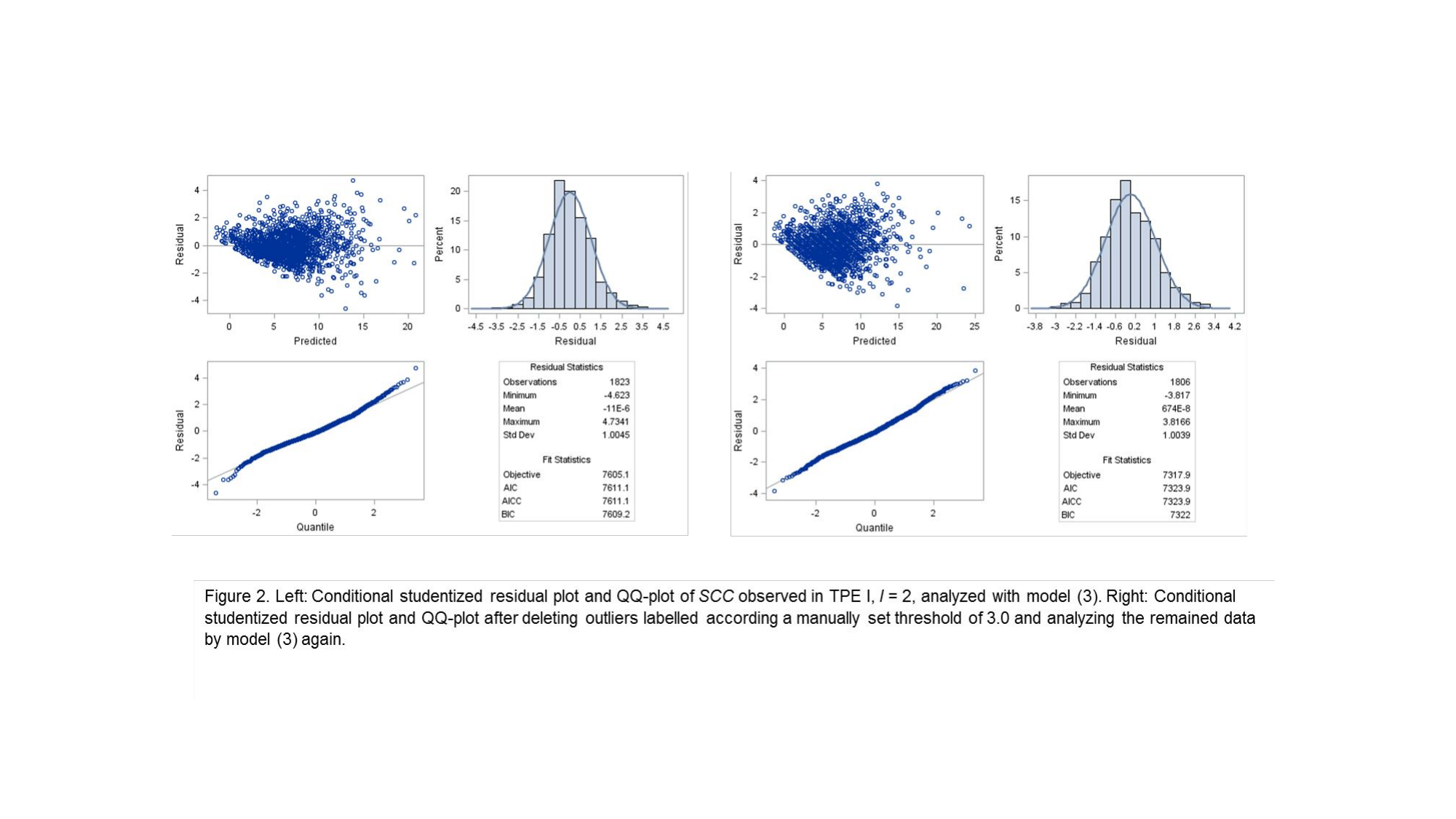

## Slide 3
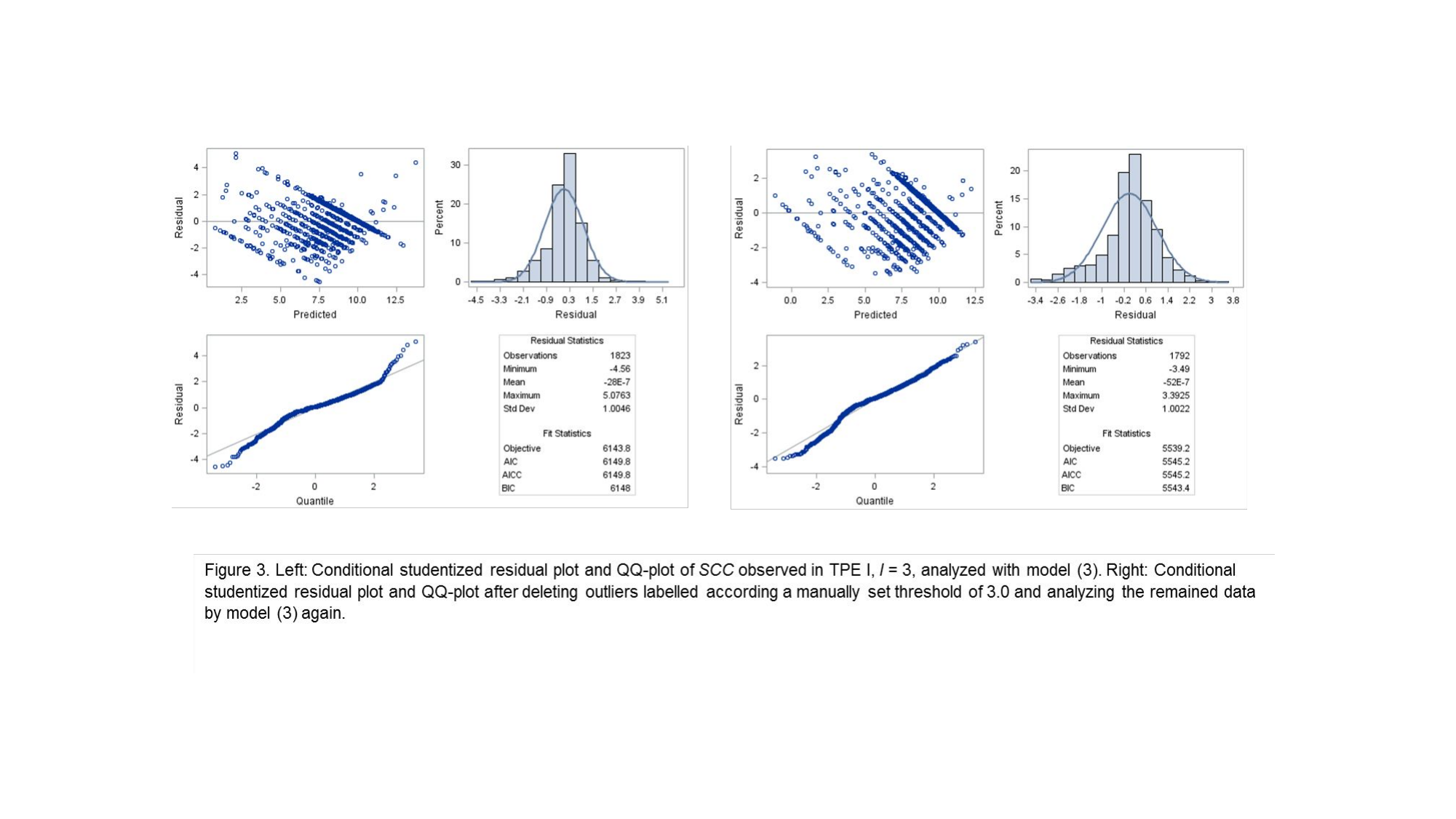

## Slide 4
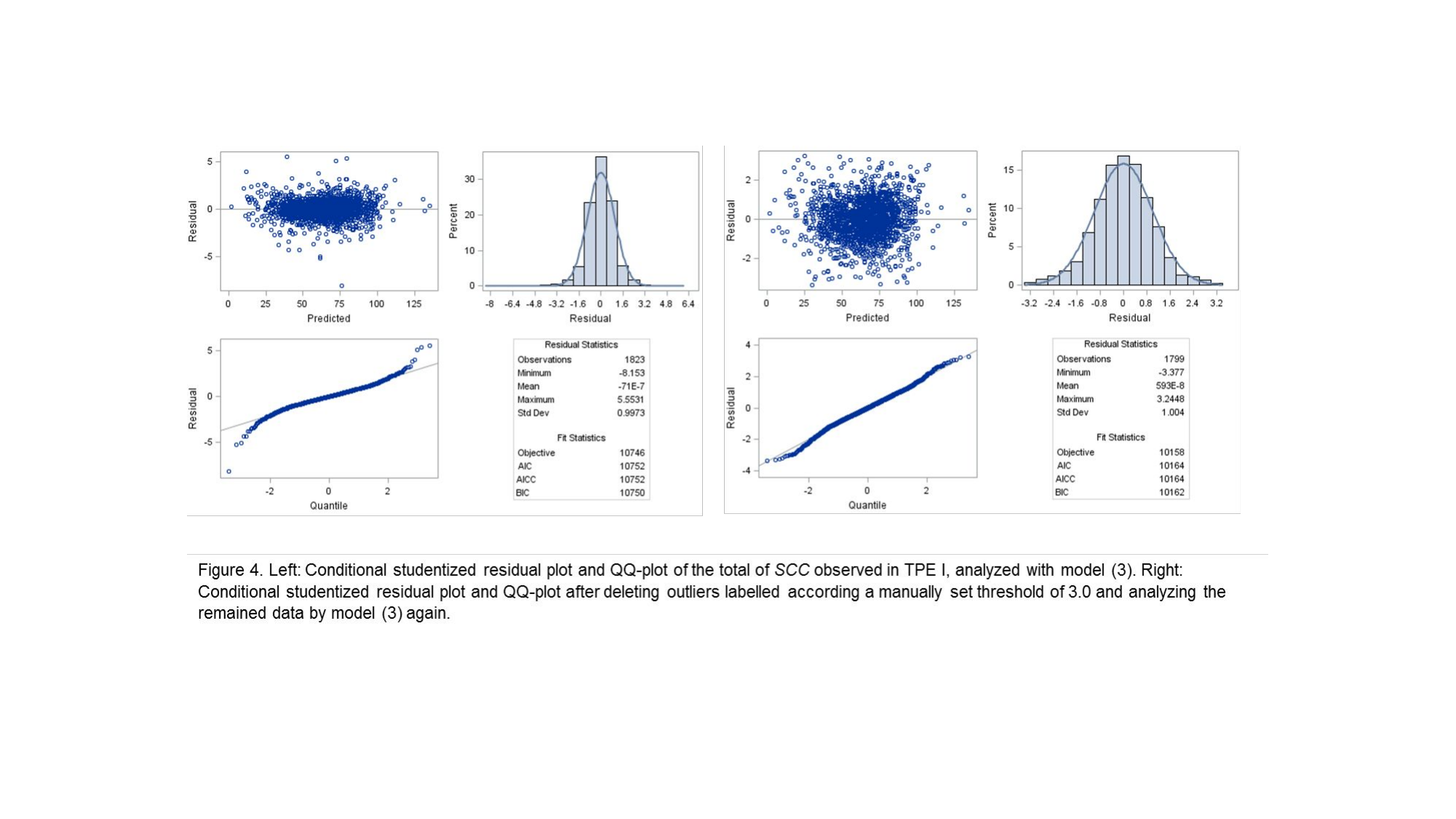

## Slide 5
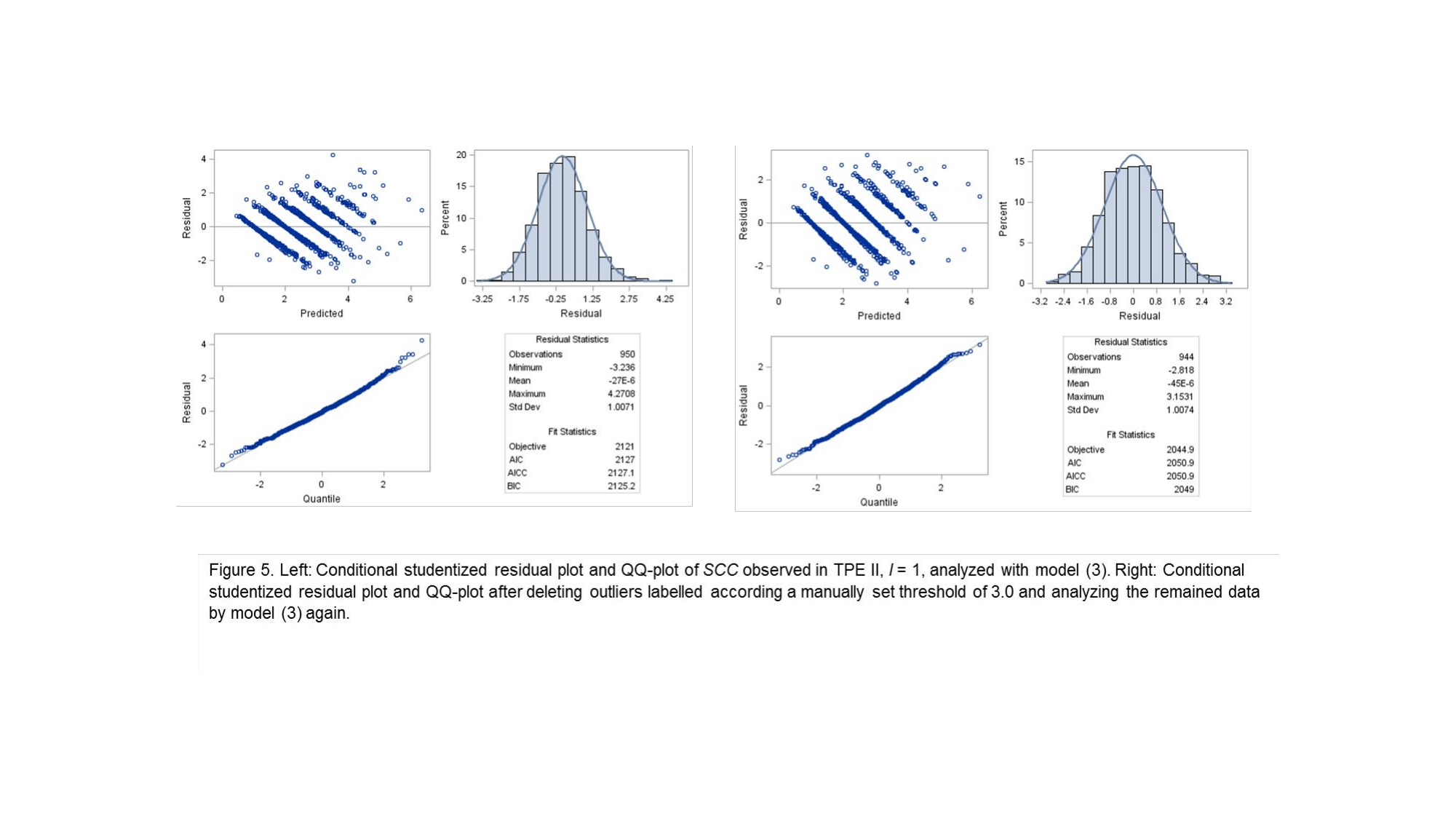

## Slide 6
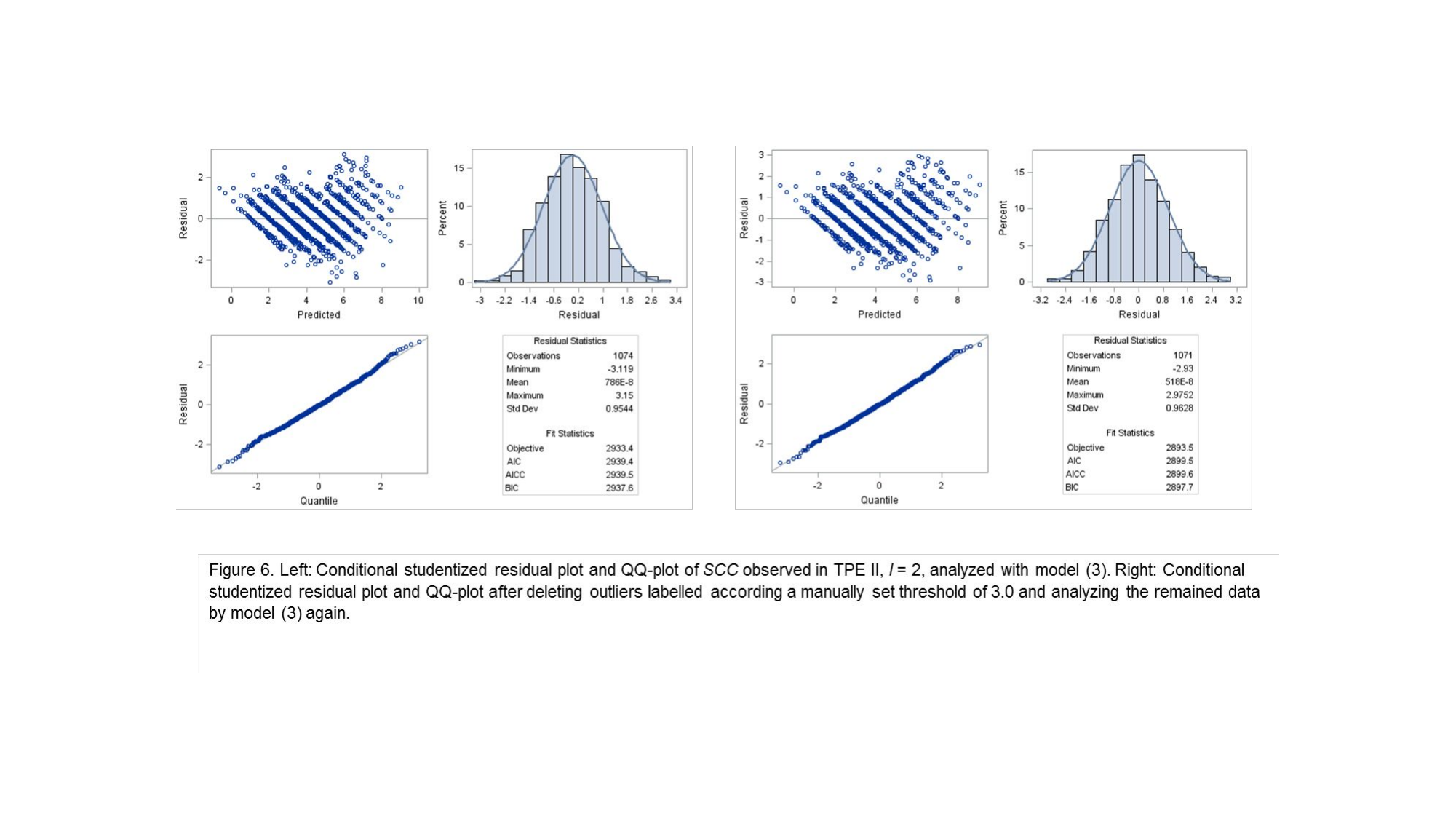

## Slide 7
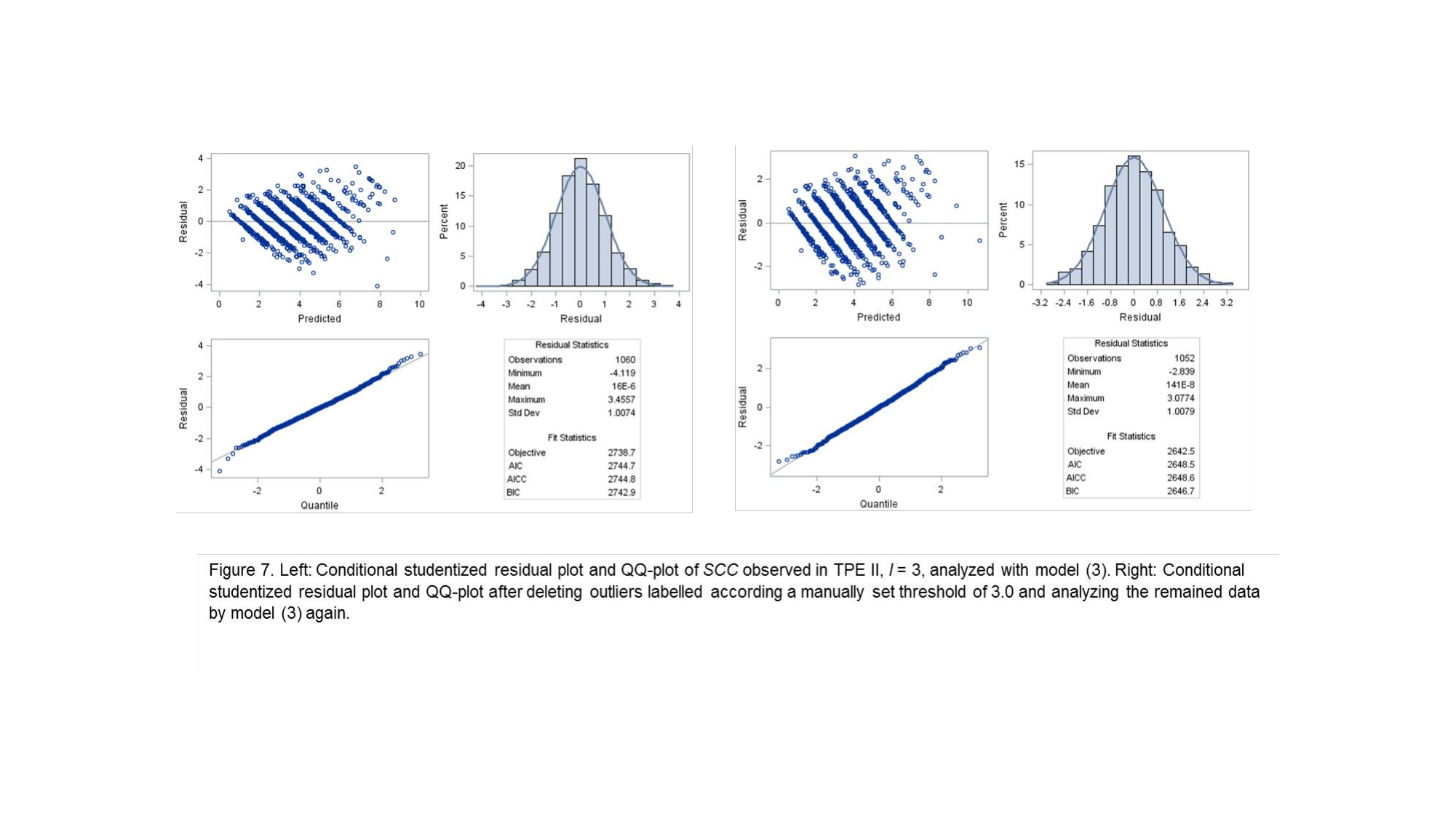

## Slide 8
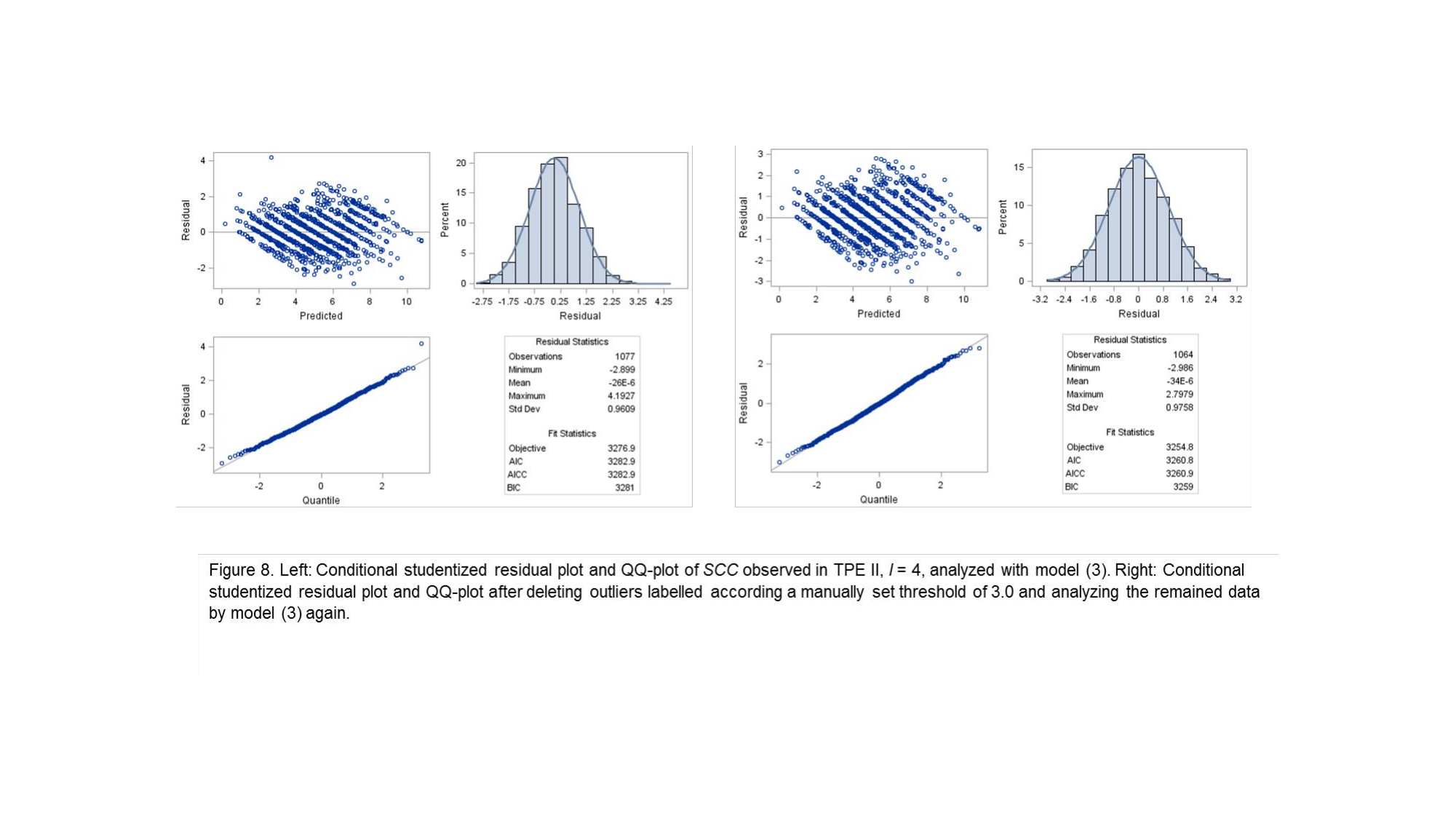

## Slide 9
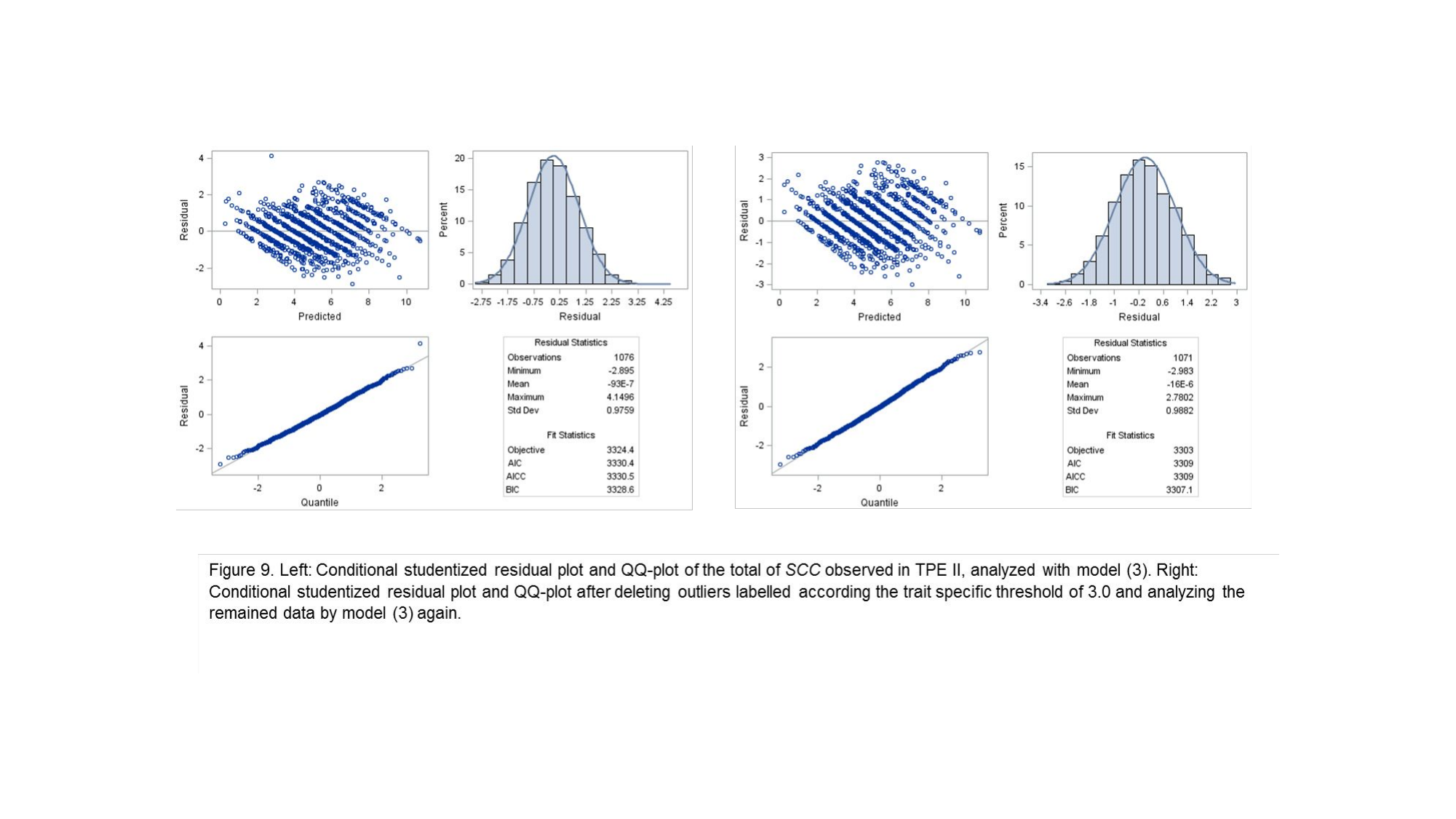

## Slide 10
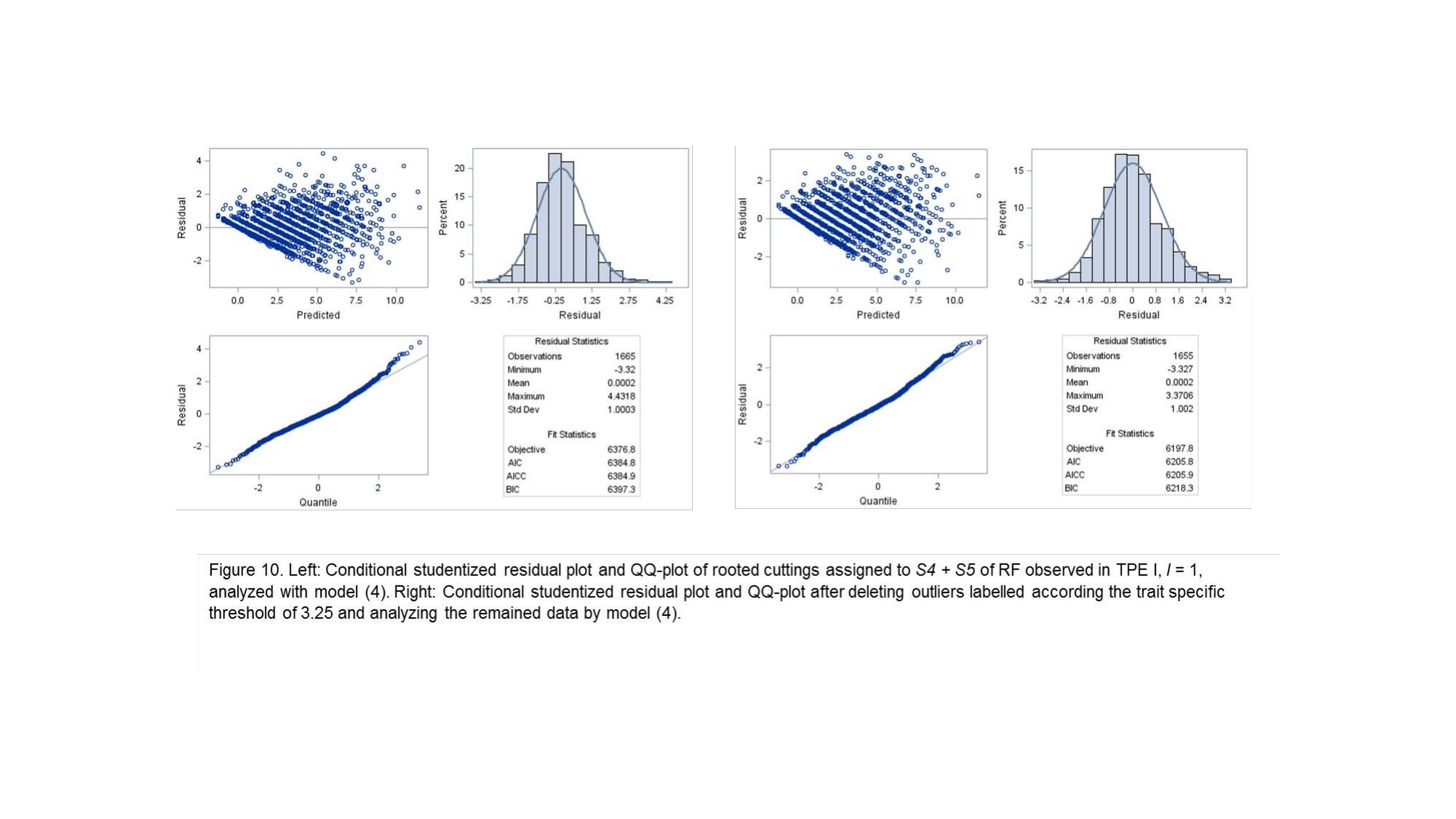

## Slide 11
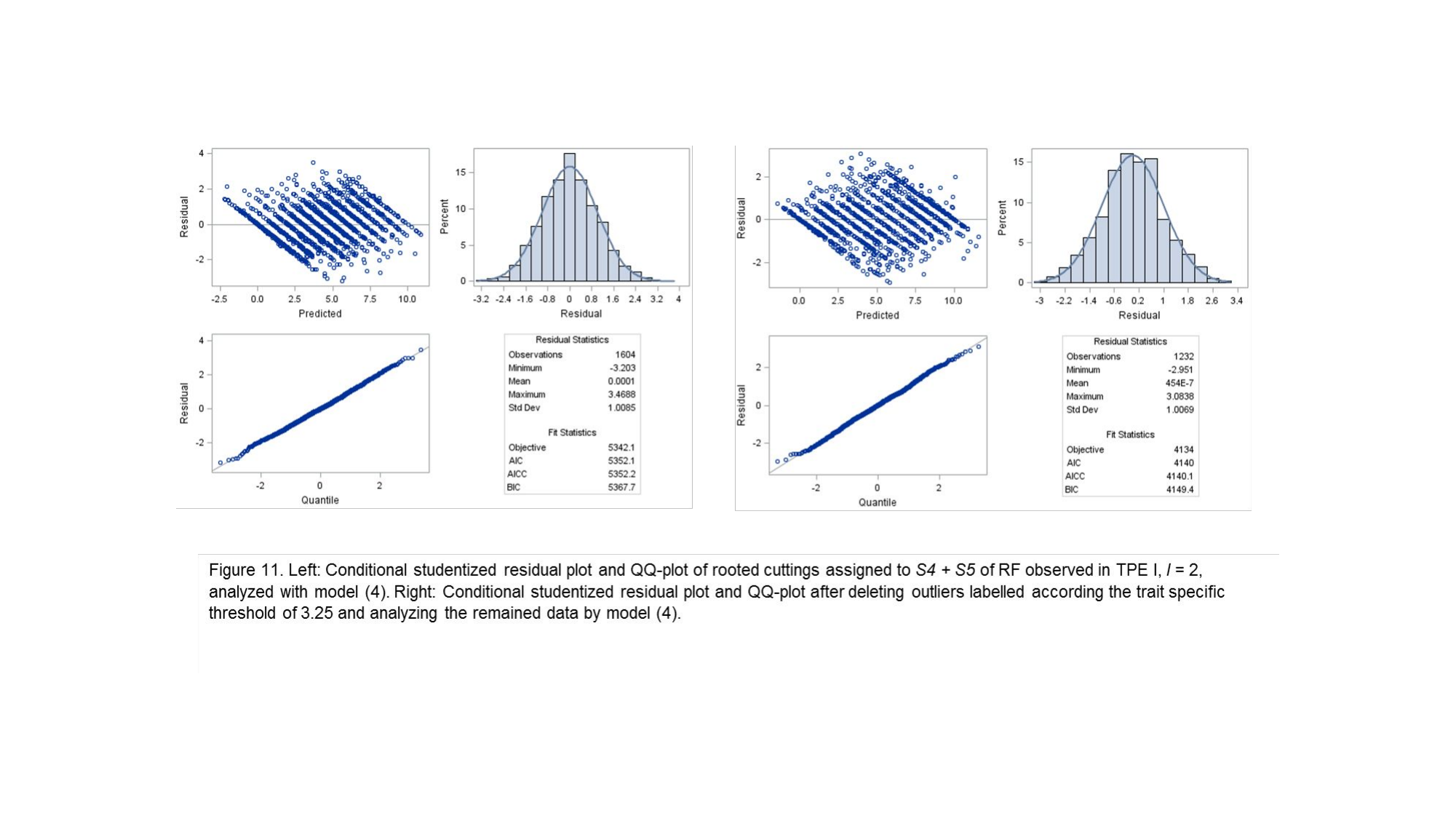

## Slide 12
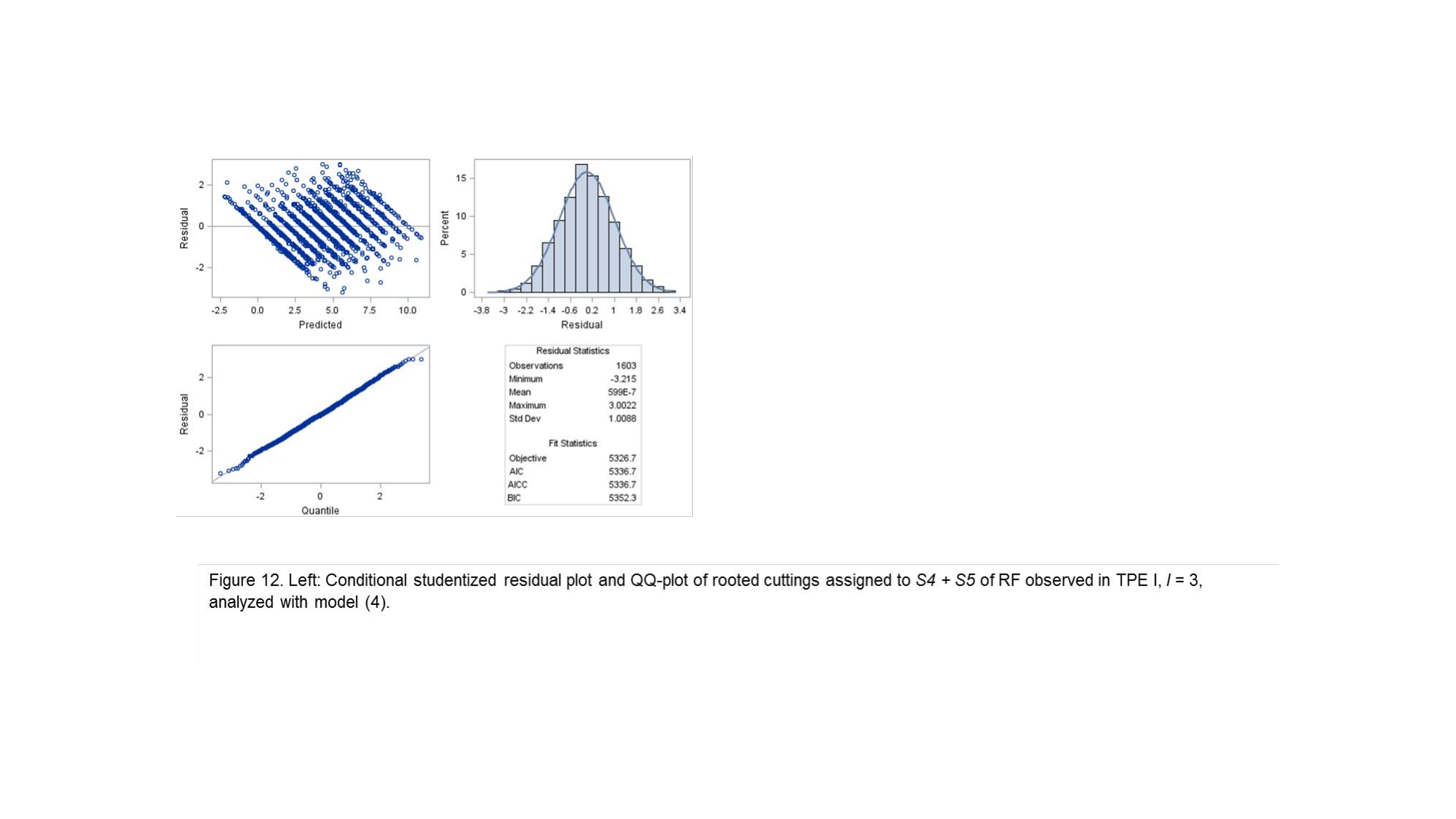

## Slide 13
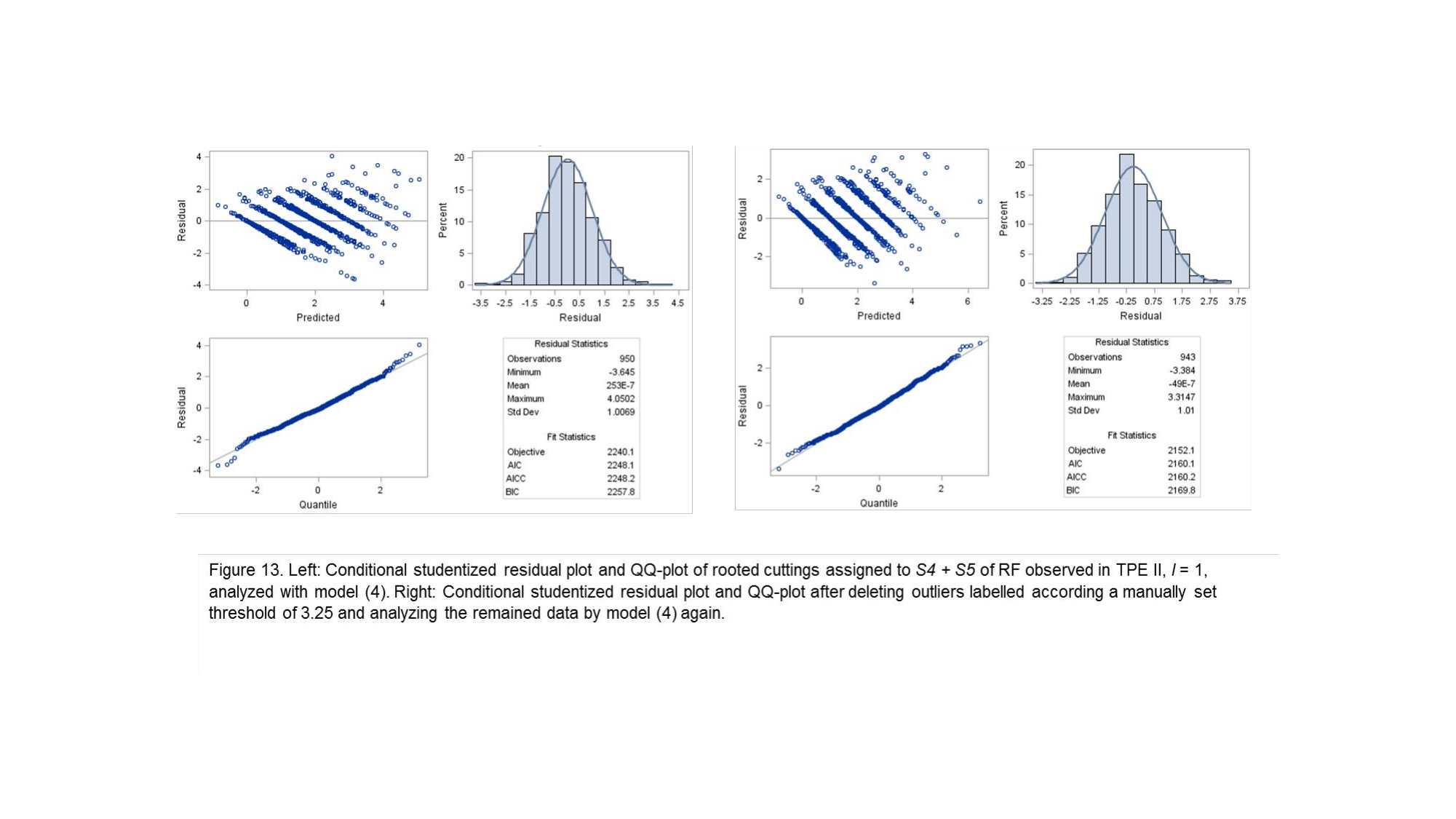

## Slide 14
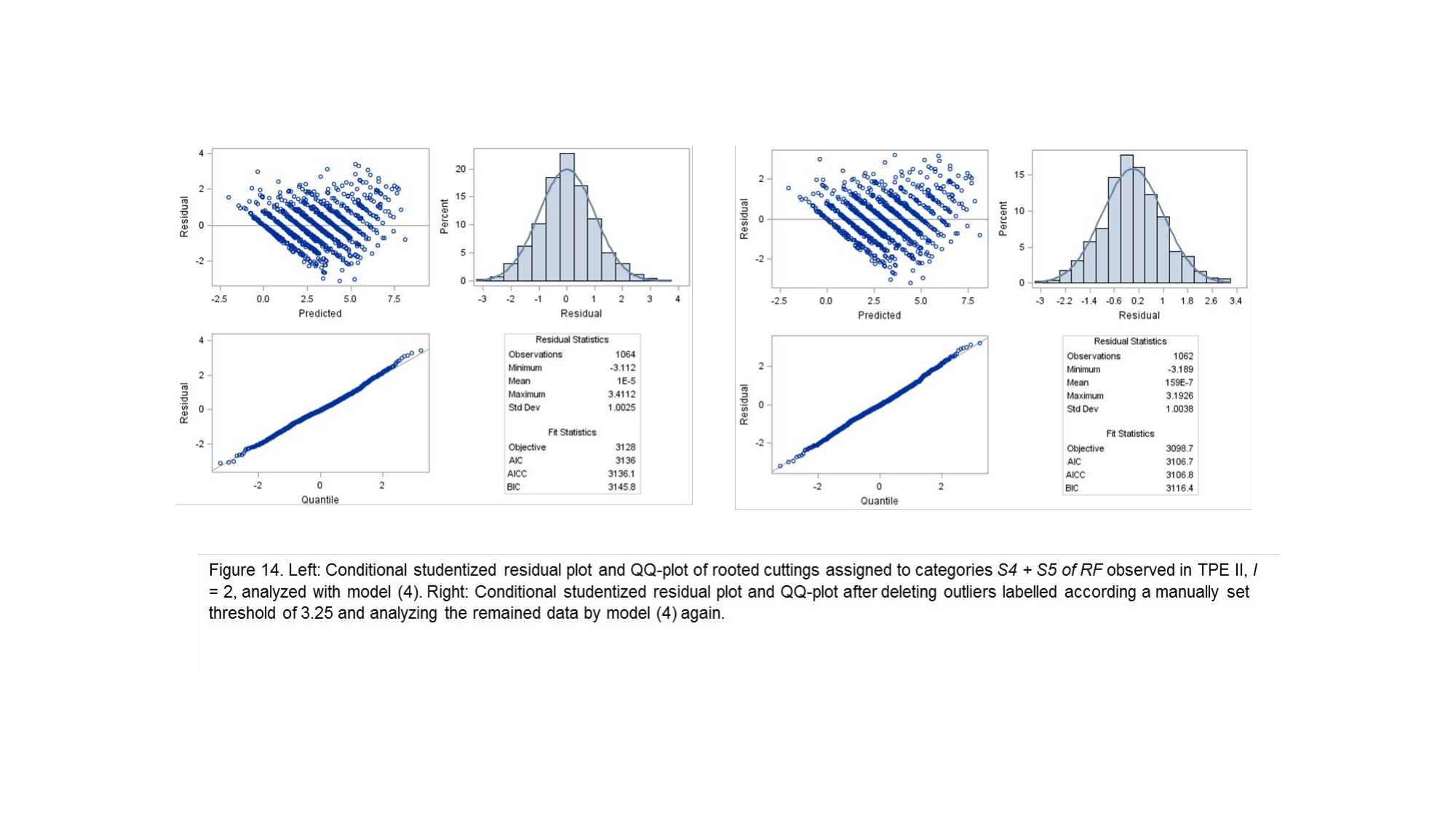

## Slide 15
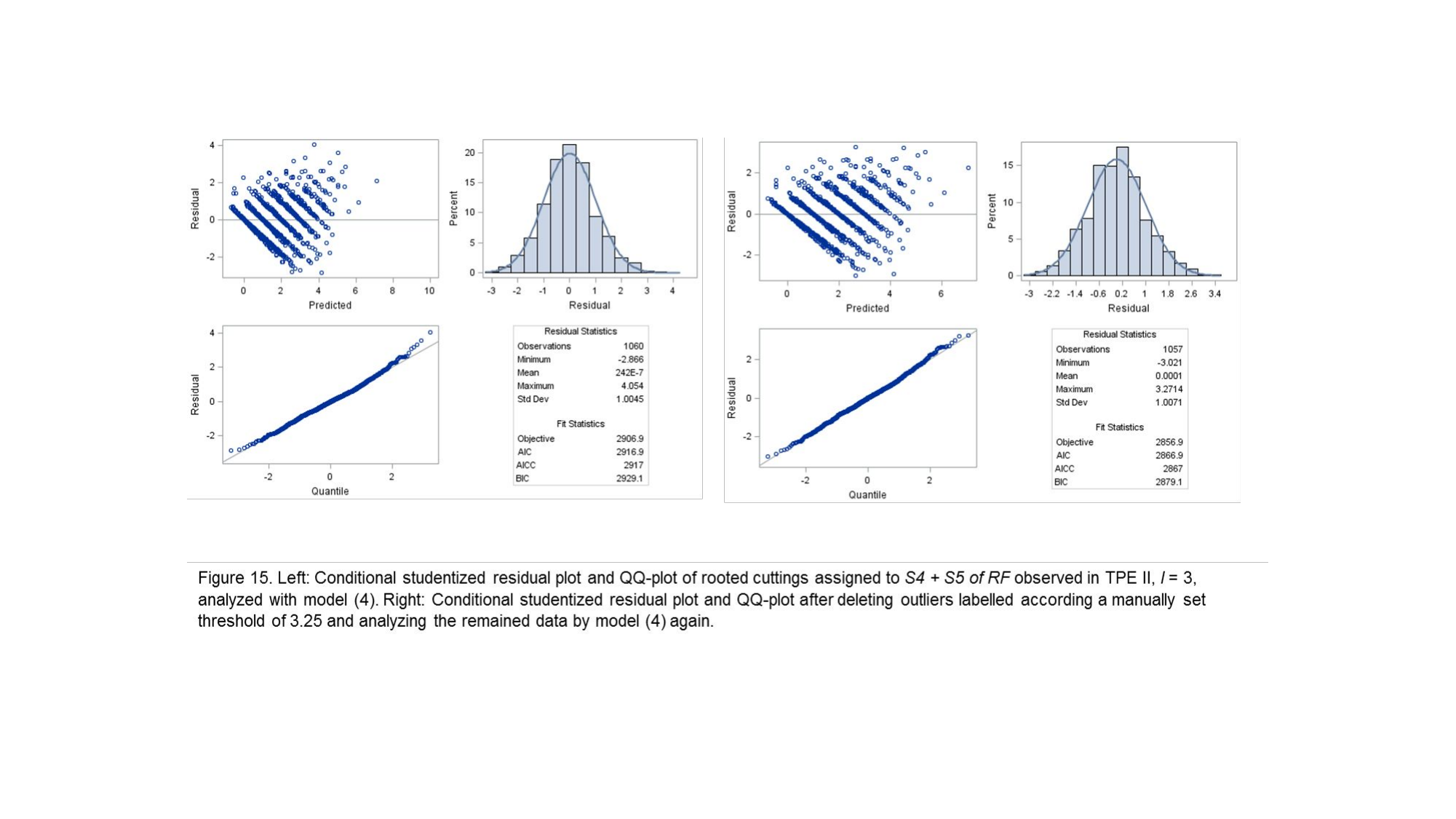

## Slide 16
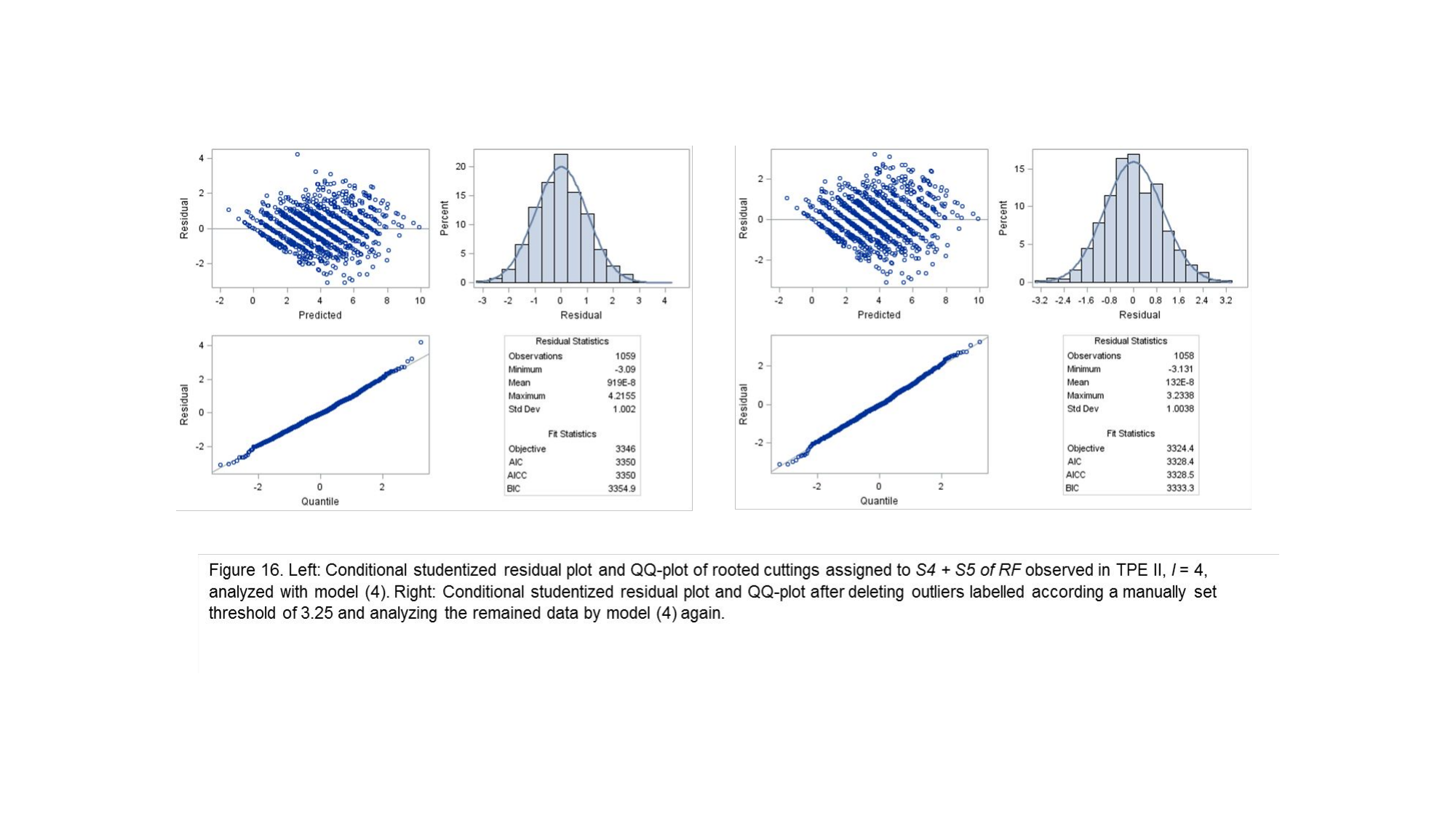

## Slide 17
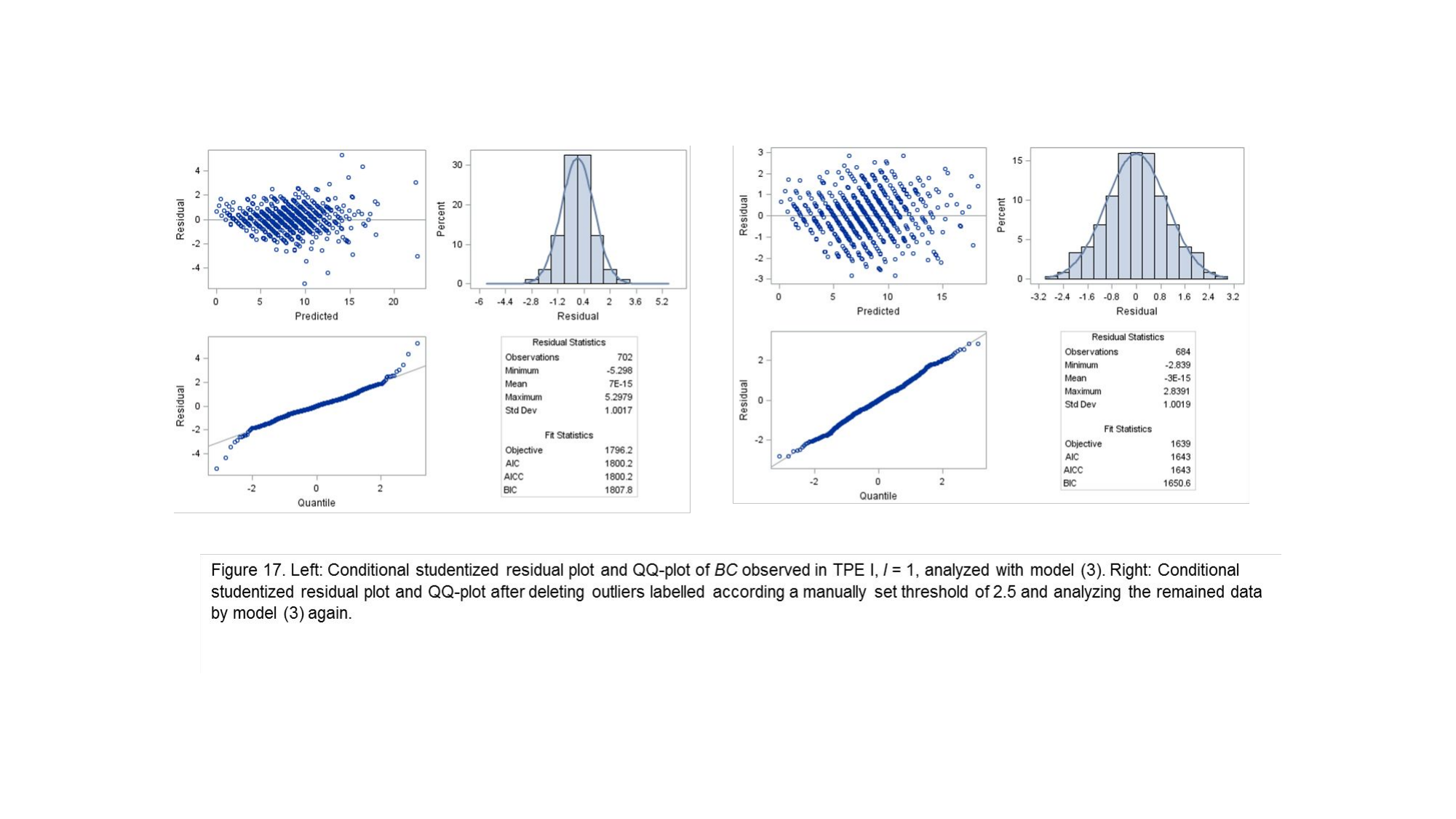

## Slide 18
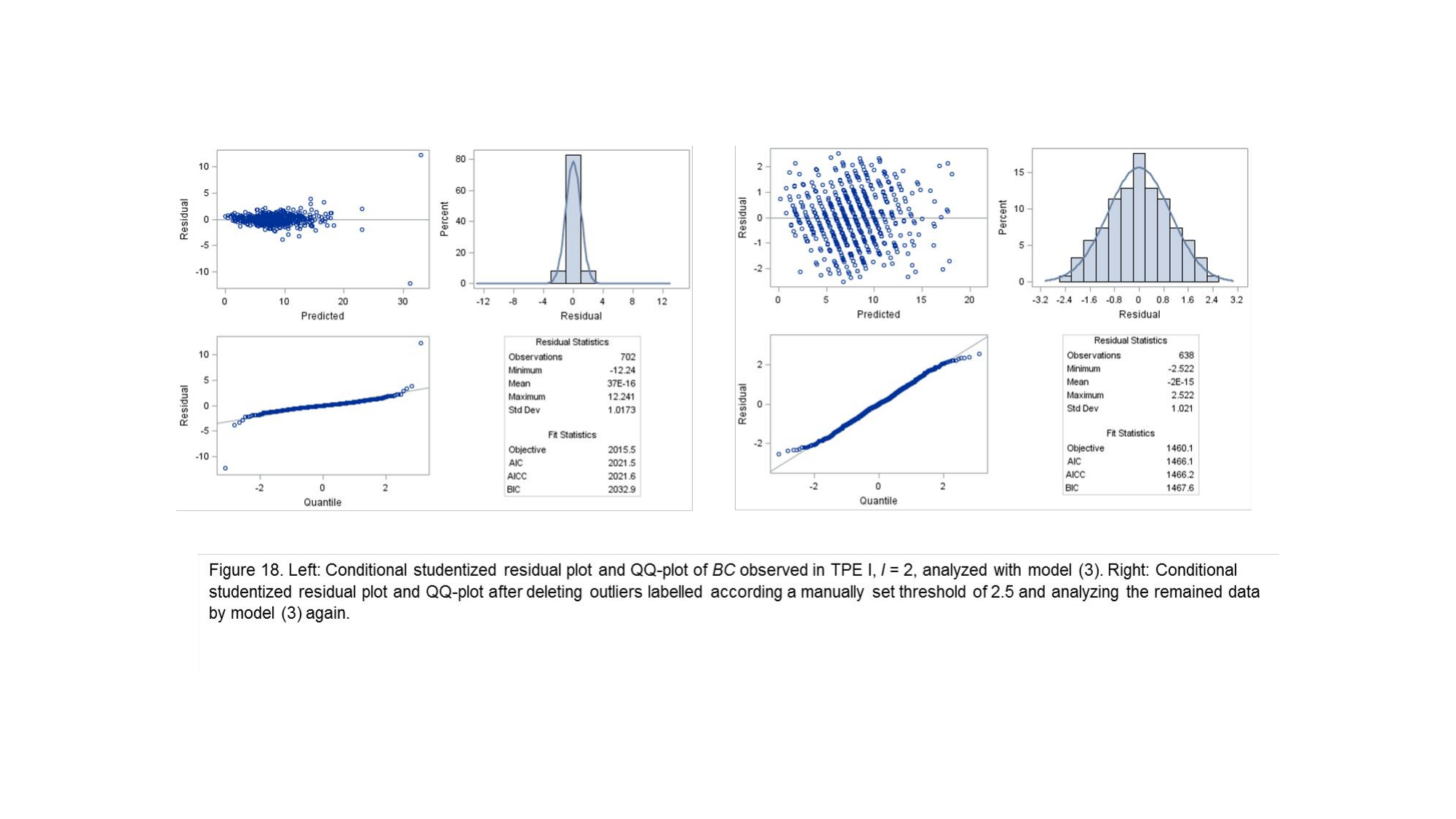

## Slide 19
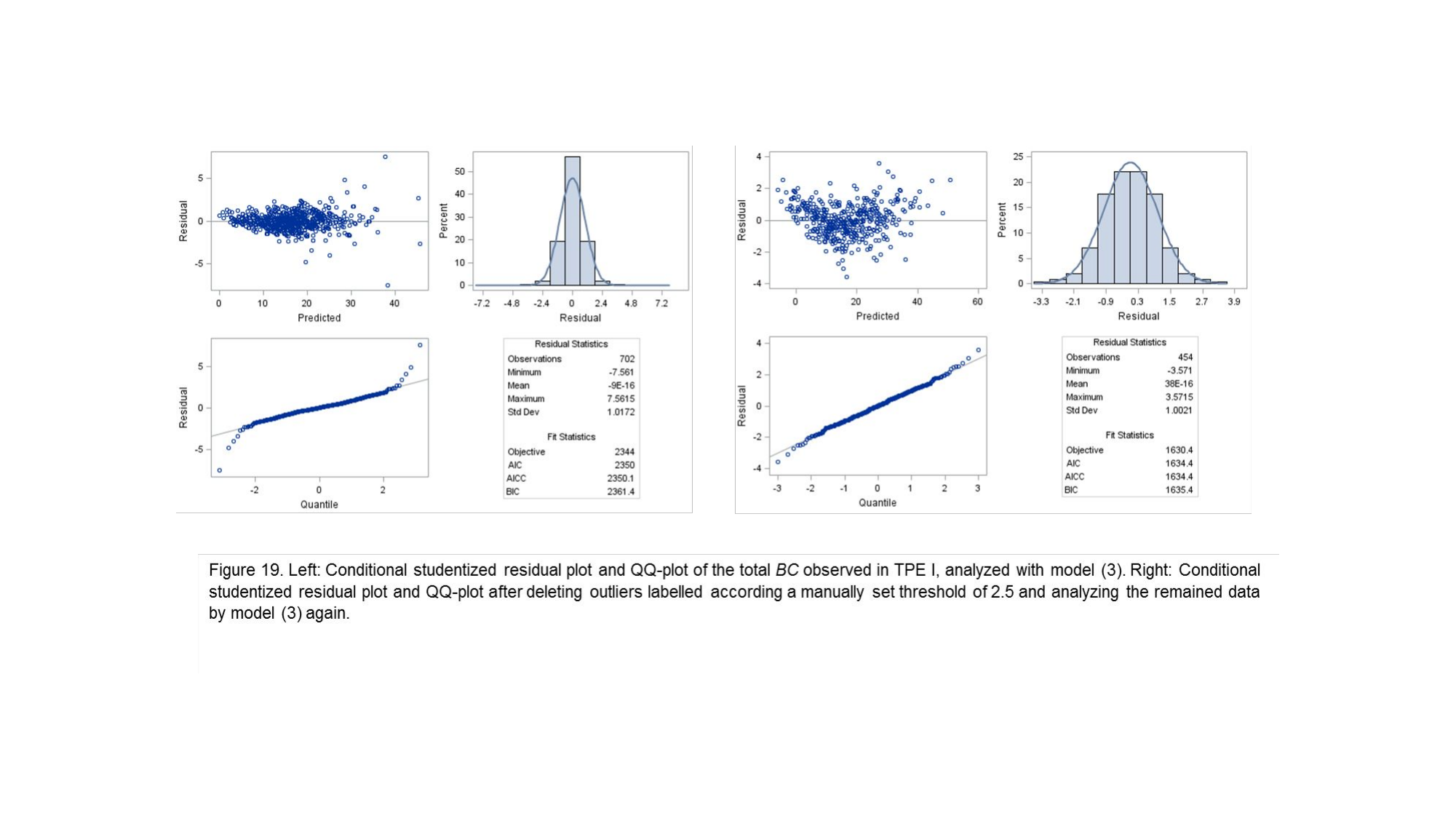

## Slide 20
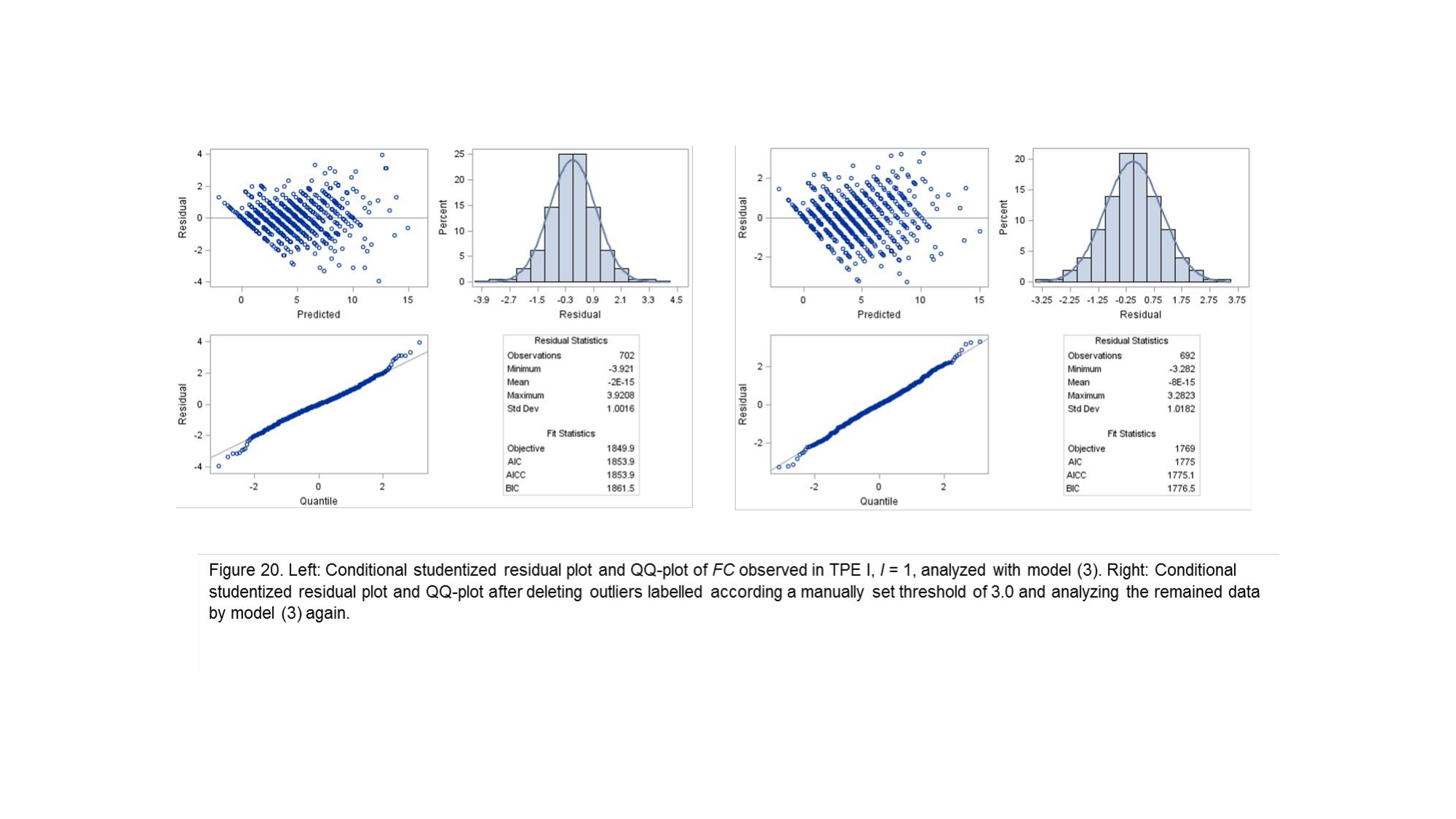

## Slide 21
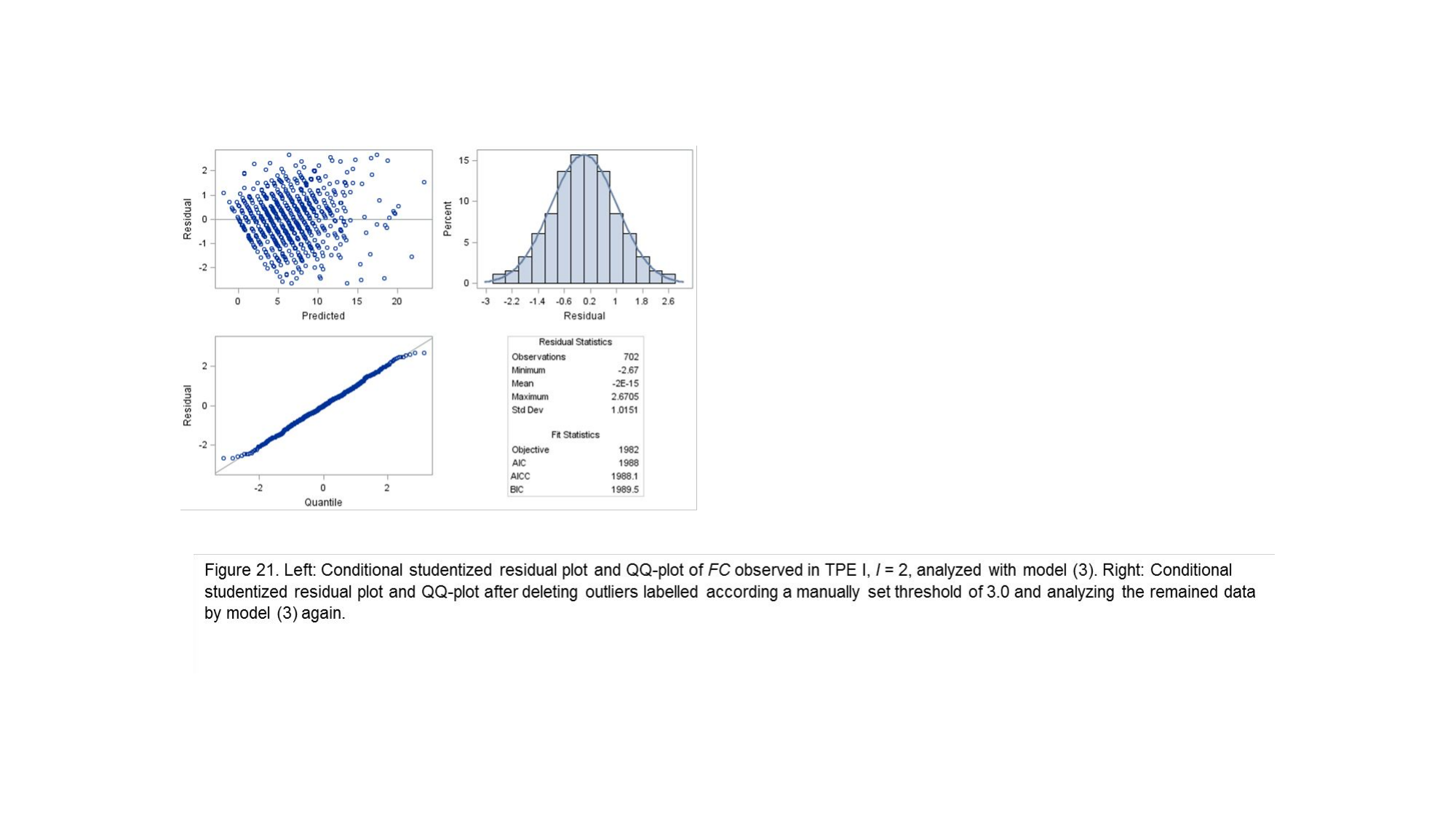

## Slide 22
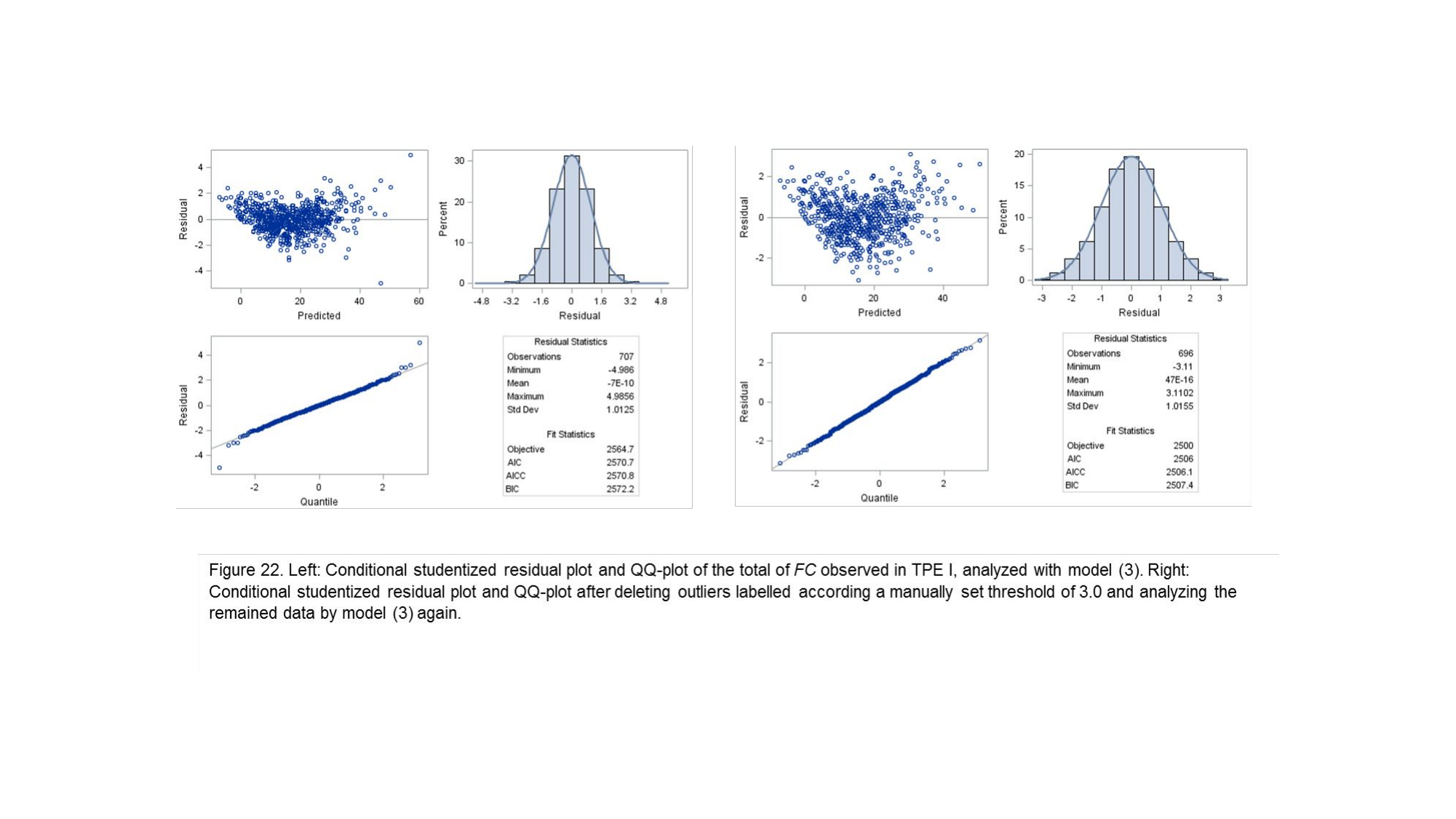

Supplement: Supplementary Figures 1–22 [file hortres20174-s1.ppt]
