# Supplementary material for: Selection for production-related traits in Pelargonium zonale: improved design and analysis make all the difference
Source: Hortic Res. 2017 Feb 22;4:17004–. doi: 10.1038/hortres.2017.4 (PMC5321157; doi:10.1038/hortres.2017.4)
Supplement: Supplementary Tables [file hortres20174-s3.pdf]

Variance components of repeated measurement analysis including serial correlation of observations

Table 1: Variance components obtained for repeated measurement analysis of stem cutting count (SCC) in TPE I by considering an unstructured variance-covariance structure for serial correlations of observations of T.REP.IB and T.REP.COL; T time effect, T.REP replicate effect, GEN genotype effect, T.GEN genotype time interaction, T.REP.IB row effect, T.REP.COL column effect, T.REP.IB.PAIR residual error

**Effect**	***l*, the time-point of observation**	**Variance parameter**	**Estimate**
**T**			2.0746
**T.REP**			0.1864
**GEN**			2.4271
**T.GEN**			0.6684
**T.REP.IB**	**1**	**VAR 1**	0
	**2**	**VAR 2**	0.0034
	**3**	**VAR 3**	0.0001
		**COV 2 - 1**	0.0001
		**COV 3 - 1**	0
		**COV 3 - 2**	0.0004
**T.REP.COL**	**1**	**VAR 1**	0.3638
	**2**	**VAR 2**	0.0834
	**3**	**VAR 3**	0.0076
		**COV 2 - 1**	0.0948
		**COV 3 - 1**	0.0044
		**COV 3 - 2**	0.0222
**T.REP.IB.PAIR**	**1**	**VAR 1**	7.2480
	**2**	**VAR 2**	10.8184
	**3**	**VAR 3**	2.6309
		**COV 2 - 1**	2.4872
		**COV 3 - 1**	0.3703
		**COV 3 - 2**	0.4035

Table 2: Variance components obtained for repeated measurement analysis of rooted stem cutting counts assigned to (*S4* + *S5*) of root formation (RF) in TPE I by considering a compound symmetry variance-covariance structure for serial correlations of observations of observations for T.REP.IB and T.REP.COL; T time effect, T.REP replicate effect, GEN genotype effect, T.GEN genotype time interaction, T.REP.IB row effect, T.REP.COL column effect, RTABLE rooting table effect, RTABLE.TRAY tray effect, T.REP.IB.PAIR residual error		
**Effect**	**Variance parameter**	**Estimate**
**T**		0.4980
**T.REP**		0.9921
**GEN**		1.7444
**T.GEN**		0.7786
**T.REP.IB**	**VAR**	0.0341
	**COV**	0
**T.REP.COL**	**VAR**	0.1657
	**COV**	0.0254
**T.REP.IB.COL**	**VAR**	0.5957
**T.RTABLE**	**VAR**	0.2608
**T.RTABLE.TRAY**	**VAR**	0.6208
**T.REP.IB.PAIR**	**VAR**	4.8506

Table 3: Variance components obtained for repeated measurement analysis of flower count (FC) in TPE I by considering an unstructured variance-covariance structure for serial correlations of observations for T.REP.IB and T.REP.COL; T time effect, T.REP replicate effect, GEN genotype effect, T.GEN genotype time interaction, T.REP.IB row effect, T.REP.COL column effect, T.REP.IB.PAIR residual error			
**Effect**	***l*, the time-point of observation**	**Variance parameter**	**Estimate**
**T**			1.9184
**T.REP**			0
**GEN**			2.8560
**T.GEN**			2.5310
**T.REP.IB**	**1**	**VAR 1**	0.0010
	**2**	**VAR 2**	0.0002
		**COV 2 - 1**	0.0004
**T.REP.COL**	**1**	**VAR 1**	0.3454
	**2**	**VAR 2**	0.9113
		**COV 2 - 1**	0.1885
**T.REP.IB.PAIR**	**1**	**VAR 1**	4.3297
	**2**	**VAR 2**	8.7000
		**COV 2 - 1**	1.6435

**Table 4: Variance components obtained for repeated measurement analysis of branch count (BC) in TPE I by considering an unstructured variance-covariance structure for serial correlations of observations for T.REP.IB and T.REP.COL; T time effect, T.REP replicate effect, GEN genotype effect, T.GEN genotype time interaction, T.REP.IB row effect, T.REP.COL column effect, T.REP.IB.PAIR residual error**			
**Effect**	***l*, the time-point of observation**	**Variance parameter**	**Estimate**
**T**			0
**T.REP**			0
**GEN**			5.6121
**T.GEN**			0
**T.REP.IB**	**1**	**VAR 1**	0.2565
**T.REP.IB**	**2**	**VAR 2**	1.1762
		**COV 2 - 1**	0.3927
**T.REP.COL**	**1**	**VAR 1**	0.2853
	**2**	**VAR 2**	0.3329
		**COV 2 - 1**	0.1653
**T.REP.IB.PAIR**	**1**	**VAR 1**	6.2738
	**2**	**VAR 2**	6.8059
		**COV 2 - 1**	6.2940

**Table 5: Variance components obtained for repeated measurement analysis of stem cutting count (SCC) in TPE II by considering an unstructured variance-covariance structure for serial correlations of observations for T.REP.IB and T.REP.COL; T time effect, T.REP replicate effect, GEN genotype effect, T.GEN genotype time interaction, T.REP.IB row effect, T.REP.COL column effect, T.REP.IB.PAIR residual error**			
**effect**	***l*, the time-point of observation**	**Variance parameter**	**Estimate**
**T**			1.1128
**T.REP**			0.5604
**GEN**			0.1685
**T.GEN**			0.0042
**T.REP.IB**	**1**	**VAR 1**	0
	**2**	**VAR 2**	0.0013
	**3**	**VAR 3**	0.0011
	**4**	**VAR 4**	0.0062
		**COV 2 - 1**	0
		**COV 3 - 1**	0
		**COV 3 - 2**	0.0007
		**COV 4 - 1**	0
		**COV 4 - 2**	0.0015
		**COV 4 - 3**	0.0015
**T.REP.COL**	**1**	**VAR 1**	0.0032
	**2**	**VAR 2**	0.0254
	**3**	**VAR 3**	0.0295
	**4**	**VAR 4**	0.0450
		**COV 2 - 1**	0.0023
		**COV 3 - 1**	0.0027
		**COV 3 - 2**	-0.0004
		**COV 4 - 1**	0.0032
		**COV 4 - 2**	0.0103
		**COV 4 - 3**	0.0225
**T.REP.IB.PAIR**	**1**	**VAR 1**	1.0016
	**2**	**VAR 2**	2.4652
	**3**	**VAR 3**	1.6834
	**4**	**VAR 4**	4.1452
		**COV 2 - 1**	-0.1565
		**COV 3 - 1**	0.0763
		**COV 3 - 2**	0.4756
		**COV 4 - 1**	0.1136
		**COV 4 - 2**	1.1030
		**COV 4 - 3**	0.7996

**Table 6: Variance components obtained for repeated measurement analysis of rooted stem cutting counts assigned to (*S4* + *S5*) of root formation (RF) in TPE II by considering an unstructured variance-covariance structure for serial correlations of observations of observations for T.REP.IB and T.REP.COL; T time effect, T.REP replicate effect, GEN genotype effect, T.GEN genotype time interaction, T.REP.IB row effect, T.REP.COL column effect, RTABLE rooting table effect, RTABLE.TRAY tray effect, T.REP.IB.PAIR residual error**			
**Effect**	***l*, the time-point of observation**	**Variance parameter**	**Estimate**
**T**			0.9205
**T.REP**			0.3616
**GEN**			0.3579
**T.GEN**			0.1303
**T.REP.IB**	**1**	**VAR 1**	0
	**2**	**VAR 2**	0.0030
	**3**	**VAR 3**	0.0023
	**4**	**VAR 4**	0.0267
		**COV 2 - 1**	0
		**COV 3 - 1**	0
		**COV 3 - 2**	0.0025
		**COV 4 - 1**	0
		**COV 4 - 2**	0.0055
		**COV 4 - 3**	0.0045
**T.REP.COL**	**1**	**VAR 1**	0.0006
	**2**	**VAR 2**	0.0958
	**3**	**VAR 3**	0.0172
	**4**	**VAR 4**	0.1224
		**COV 2 - 1**	-0.0002
		**COV 3 - 1**	0.0007
		**COV 3 - 2**	0.0201
		**COV 4 - 1**	0.0020
		**COV 4 - 2**	0.0555
		**COV 4 - 3**	0.0213
**T.RTABLE**			1.4165
**T.RTABLE.TRAY**			0.0896
**T.REP.IB.PAIR**	**1**	**VAR 1**	1.5231
	**2**	**VAR 2**	2.4318
	**3**	**VAR 3**	2.9093
	**4**	**VAR 4**	3.6675
		**COV 2 - 1**	0.2605
		**COV 3 - 1**	0.2819
		**COV 3 - 2**	0.7530
		**COV 4 - 1**	0.3815
		**COV 4 - 2**	0.6271
		**COV 4 - 3**	0.8815
